# Efficacy and safety of Chinese medicine injection combined with concurrent chemoradiotherapy in the treatment of esophageal cancer: a Bayesian network meta-analysis

**DOI:** 10.3389/fmed.2025.1643598

**Published:** 2025-10-14

**Authors:** Jiacheng Wang, Hongbin Xi, Xuewei Chen, Yingqian Xin, Fengqin Wei

**Affiliations:** ^1^College of Traditional Chinese Medicine, Shandong University of Traditional Chinese Medicine, Jinan, China; ^2^Department of Traditional Chinese Medicine Classics, Tai’an Hospital of Traditional Chinese Medicine, Tai’an, China

**Keywords:** esophageal cancer, Chinese medicine injection, concurrent chemoradiotherapy, network meta-analysis, complementary medicine

## Abstract

**Background:**

Esophageal cancer (EC) is a significant global health concern. Chinese medicine injections (CMIs) are widely utilized as adjunctive therapies for EC. This network meta-analysis (NMA) aimed to compare the efficacy and safety of various CMIs in combination with concurrent chemoradiotherapy (CCRT) for the treatment of EC.

**Methods:**

Relevant randomized controlled trials (RCTs) were comprehensively searched in eight electronic databases until August 2024. The quality of eligible RCTs was assessed via the Cochrane Risk of Bias tool (RoB 2.0). Bayesian NMA was conducted through R 4.2.1 and Stata 15.1, with treatment regimens ranked based on the surface under the cumulative ranking curve (SUCRA). The quality of evidence was evaluated using CINeMA.

**Results:**

54 studies encompassing 4,201 patients and 13 types of CMIs were included. Astragalus polysaccharide injection (HQDT) combined with CCRT (SUCRA: 86.7%) ranked highest for improving clinical effectiveness rate. Kanglaite injection (KLT) combined with CCRT (SUCRA: 85.1%; 90.1%) was optimal for enhancing performance status and one-year survival rate. Kangai injection (KA) combined with CCRT (SUCRA: 97.2%) achieved the greatest improvement in CD3^+^ levels. Aidi injection (AD) combined with CCRT (SUCRA: 99.9, 99.9%) was most effective in increasing CD4^+^ and CD8^+^ levels, while Fufangkushen injection (FFKS) combined with CCRT (SUCRA: 99.9%) yielded the greatest improvement in the CD4^+^/CD8^+^ ratio. Based on descriptive statistics, all regimens demonstrated favorable safety profiles, with no serious adverse events (AEs) reported.

**Conclusion:**

CMIs combined with CCRT appear to provide superior therapeutic efficacy over CCRT alone in the treatment of EC. In particular, HQDT, KLT, KA, AD, and FFKS exhibited the most pronounced benefits across key clinical outcomes. Nevertheless, the findings shall be validated in multicenter, better-designed RCTs.

**Systematic review registration:**

The PRISMA registration details for this study can be found at: https://www.crd.york.ac.uk/PROSPERO/view/CRD42024574242.

## 1 Introduction

Esophageal cancer (EC) ranks seventh among cancers in terms of mortality and is the eleventh most frequently diagnosed malignancy worldwide. In 2022, approximately 511,000 new cases and 445,000 deaths were reported globally (1). Most cases are diagnosed at advanced stages with distant metastasis because there are no early clinical symptoms (2, 3). Despite comprehensive treatment, including surgery, the five-year survival rate is typically below 20% (4, 5). The disease burden is particularly pronounced in Asia, where an estimated 383,000 new cases and 329,000 deaths were reported in 2022, accounting for roughly 75% of the global incidence and mortality (6). With population aging and the persistent prevalence of major risk factors, including tobacco and alcohol consumption, elevated body mass index (BMI), and unhealthy dietary habits, the medical burden of EC is expected to escalate further (7). By 2040, it is projected that over 900,000 people worldwide will die from EC, which poses a significant challenge to public health systems (8).

Currently, concurrent chemoradiotherapy (CCRT) plays an important role in patients with advanced EC, not only as adjuvant therapy but also as definitive treatment (9). The 2024 National Comprehensive Cancer Network (NCCN) guidelines for EC (10) recommend paclitaxel plus carboplatin in combination with radiotherapy as the preferred regimen. This approach has been shown to improve surgical resection rates in advanced EC, as well as overall survival (OS) and disease-free survival (DFS) (11, 12). Nonetheless, the therapeutic efficacy remains limited, and the prognosis is often poor. In patients receiving paclitaxel-carboplatin-based definitive chemoradiotherapy (dCRT), the local recurrence rate can reach 47.9%, with 35.2% experiencing both local recurrence and distant metastases (13). Moreover, the synergistic effects of chemoradiotherapy can lead to cumulative toxicity, causing long-term damage and markedly increasing the incidence of adverse events (AEs) such as myelosuppression, gastrointestinal reactions, and radiation-induced esophagitis, as well as raising the risk of late toxicity and postoperative mortality (14–17).

Traditional Chinese medicine (TCM) has emerged as a valuable adjunct in oncology, with demonstrated benefits in enhancing antitumor efficacy, alleviating clinical symptoms, and mitigating the toxic side effects of CCRT (18, 19). Chinese medicine injections (CMIs), an important component of TCM, ingeniously integrate TCM theories with modern pharmaceutical technology. These injections are refined by extracting active components from herbal medicines and natural products (20, 21). CMIs offer high concentrations, rapid absorption, and improved bioavailability, and have been widely applied in the treatment of non-small cell lung, breast, cervical, gastric, and colorectal cancers, among others (22–26). In TCM theory, EC falls within the category of esophageal obstruction, with a core pathogenesis involving the interlocking of phlegm and blood stasis, depletion of body fluids, and the accumulation of heat toxins—often precipitated by emotional distress and irregular diet. Early-stage EC is characterized by a sensation of obstruction on swallowing and a feeling of fullness in the chest and diaphragm, consistent with qi stagnation and phlegm accumulation. In the intermediate stage, blood stasis predominates, leading to worsening dysphagia and stabbing chest or back pain. In the late stage, patients often present with severe dysphagia to both solids and liquids, marked emaciation, and symptoms indicative of fluid depletion and internal heat accumulation. CMIs aim to regulate qi, resolve phlegm, clear heat, detoxify, and nourish qi and yin, thereby offering a promising therapeutic option for patients with EC (27). Currently, the effectiveness and safety of varied single-CMI treatments combined with chemoradiotherapy for EC have been validated (28–30). However, given the wide variety of available CMIs, comparative evidence across preparations remains insufficient, and the optimal CCRT-CMI combination for EC has not been established, posing challenges to clinical decision-making (31). Bayesian network meta-analysis (NMA) allows for the integration of direct and indirect evidence, enabling quantitative comparisons among multiple interventions and ranking their relative effectiveness and safety across diverse clinical outcomes (32). Therefore, this study aimed to employ NMA to comprehensively evaluate the efficacy and safety of different CMIs combined with CCRT in EC and offer evidence-based recommendations to guide clinical decision-making.

## 2 Methods

The present study was conducted according to the Preferred Reporting Items for Systematic Reviews and Meta-Analyses (PRISMA) guidelines, as well as the methodological requirements for network meta-analyses (NMA) (33). The checklist is presented in Supplementary material 1. Our meta-analysis was performed as per the guidelines for systematic review and meta-analysis. The protocol has been registered in the International Prospective Register of Systematic Reviews (CRD42024574242). All CMI components used in this study complied with the requirements for the reporting of plant materials as outlined in the ConPhyMP guidelines (34), including species identification, extraction procedures, and quality control (Supplementary material 2). Compliance was verified primarily through cross-checking the package inserts of CMIs approved by the National Medical Products Administration and the relevant pharmacological data reported in the included literature. None of the medicinal resources used were derived from genetic materials or endangered species subject to protection under the Nagoya Protocol or the Convention on International Trade in Endangered Species of Wild Fauna and Flora (CITES).

### 2.1 Search strategy

PubMed, Embase, Cochrane, Web of Science, China National Knowledge Infrastructure Database (CNKI), Wanfang Data, Chinese Scientific Journals Full-text Database (VIP), and Chinese Biomedical Literature Database (SinoMed) were thoroughly searched from the time of database creation through August 1, 2024. Subject headings and free text keywords were employed, with the following Medical Subject Headings (MeSH): “Esophageal Neoplasms,” “Injection,” and “randomized controlled trial (RCT).” Supplementary material 3 details the search strategy. Furthermore, a secondary search was conducted by examining references of existing systematic reviews to ensure comprehensive coverage.

### 2.2 Inclusion and exclusion criteria

The eligible studies must meet the following criteria: (1) Patients had a histopathologically confirmed diagnosis of EC, without restrictions on nationality or sex. (2) The intervention group received CMIs in combination with CCRT, including Aidi injection (AD), Fufangkushen injection (FFKS), Astragalus polysaccharides (HQDT), Kangai injection (KA), Kanglaite injection (KLT), Matrine injection (KSS), Elemene injection (LXX), Shenfu injection (SF), Shenmai injection (SM), Shenqifuzheng injection (SQFZ), Xiaoaiping injection (XAP), Xiyanping injection (XYP), and Brucea javanica oil emulsion injection (YDZYR). The control group received CCRT alone. (3) The study design was an RCT. (4) Outcomes included at least one of the following: clinical effectiveness rate, performance status, one-year survival rate, T-lymphocyte subsets (CD3+, CD4+, CD8+, CD4+/CD8 + ratio), and the incidence of AEs. The clinical effectiveness rate was calculated as per the World Health Organization (WHO) Objective Response Criteria in Solid Tumors as follows: [number of complete response (CR) patients + partial response (PR)] / total number of patients × 100%. Performance was assessed using the Karnofsky Performance Status Scale (KPS), with three categories based on KPS score changes: improvement (increase of over 10 points), stability (change of over 10 points), and decline (decrease of over 10 points). An increase in the KPS score by more than 10 points was considered a significant improvement.

The following studies were excluded: (1) Animal or cell studies, case reports, scientific experimental plans, reviews, letters, guidelines, and conference proceedings, among others;(2) Those with missing or significantly erroneous data; (3) Duplicate publications; (4) Articles with no full text.

### 2.3 Literature screening and data extraction

The retrieved studies were imported into EndNote X9. Two researchers (Wang J. C., Chen X. W.) independently screened titles and abstracts, and reviewed full texts. Any discrepancies were addressed via discussion or consultation with a third researcher (Wei F. Q.). The final data were independently extracted by the two researchers through Excel 2019, and included the first author, publication year, randomization and blinding methods, interventions and control measures, sample size, study duration, basic participant characteristics (age, tumor stage, cancer type), and outcome measures.

### 2.4 Quality assessment

The Cochrane Risk of Bias Assessment Tool (RoB 2.0) (35) was utilized to examine the quality of studies across five domains: bias originating from randomization, resulting from deviations from the intended intervention, caused by missing outcome data, in outcome measurement, and selective reporting. For every study, two reviewers (Xi H. B., Xin Y. Q.) independently assessed each aspect, classifying biases as having a “low,” “high,” or “unclear” risk. Any disputes were settled by discussing with or consulting a third researcher (Wei F. Q.). The results were detailed in the risk of bias plot.

### 2.5 Statistical analysis

The risk ratio (RR) with 95% confidence interval (CI) was utilized to quantify the clinical effectiveness rate, performance status, and one-year survival rate. Weighted mean differences (MD) with 95% CIs were used to show the rates of CD4+/CD8+, CD3+, CD4+, and CD8+. The Bayesian hierarchical random-effects model was initially fitted for comparisons of various EC treatment options due to the heterogeneity among trials (36, 37). R 4.2.1 and Stata 15.1 were utilized to generate all computations and graphics. To examine the posterior distributions of the questioned nodes, a Markov chain Monte Carlo (MCMC) simulation was conducted using Bayesian inference via R, with 500,000 iterations and 20,000 annealings, based on the theory of the likelihood function and certain presumptions (38–40). Overall model consistency was evaluated using the Deviance Information Criterion (DIC); a difference of <5 between the DIC values of the consistency and inconsistency models was interpreted as indicating satisfactory overall consistency. Convergence was assessed via the potential scale reduction factor (PSRF), with values in the range of 1.00 to <1.05 denoting adequate convergence. For outcomes involving closed loops, local inconsistency was examined using the node-splitting method. A network diagram was constructed to visualize the relationships among treatments, and publication bias was assessed using a comparison-adjusted funnel plot combined with Egger’s test (41, 42). Therapeutic ranking was determined according to the surface under the cumulative ranking curve (SUCRA), with values ranging from 0 to 1; higher SUCRA values indicated a superior ranking of EC relative to other interventions (43, 44). A league table displayed the comparative results of each pair of interventions for every outcome.

### 2.6 Evidence quality evaluation via CINeMA

The quality of evidence was evaluated using the Confidence in Network Meta-Analysis (CINeMA) framework (https://cinema.ispm.unibe.ch/). Six domains were assessed: within-study bias, reporting bias, indirectness, imprecision, heterogeneity, and incoherence. Each domain was graded as “no concerns,” “some concerns,” or “major concerns.” Overall confidence in the evidence was categorized as high, moderate, low, or very low. All included RCTs were initially rated as providing high-quality evidence. Evidence quality was downgraded if concerns were identified in any domain, with the extent of downgrading determined by the severity of the issue (45).

## 3 Results

### 3.1 Literature search and selection process

**Figure 1:**
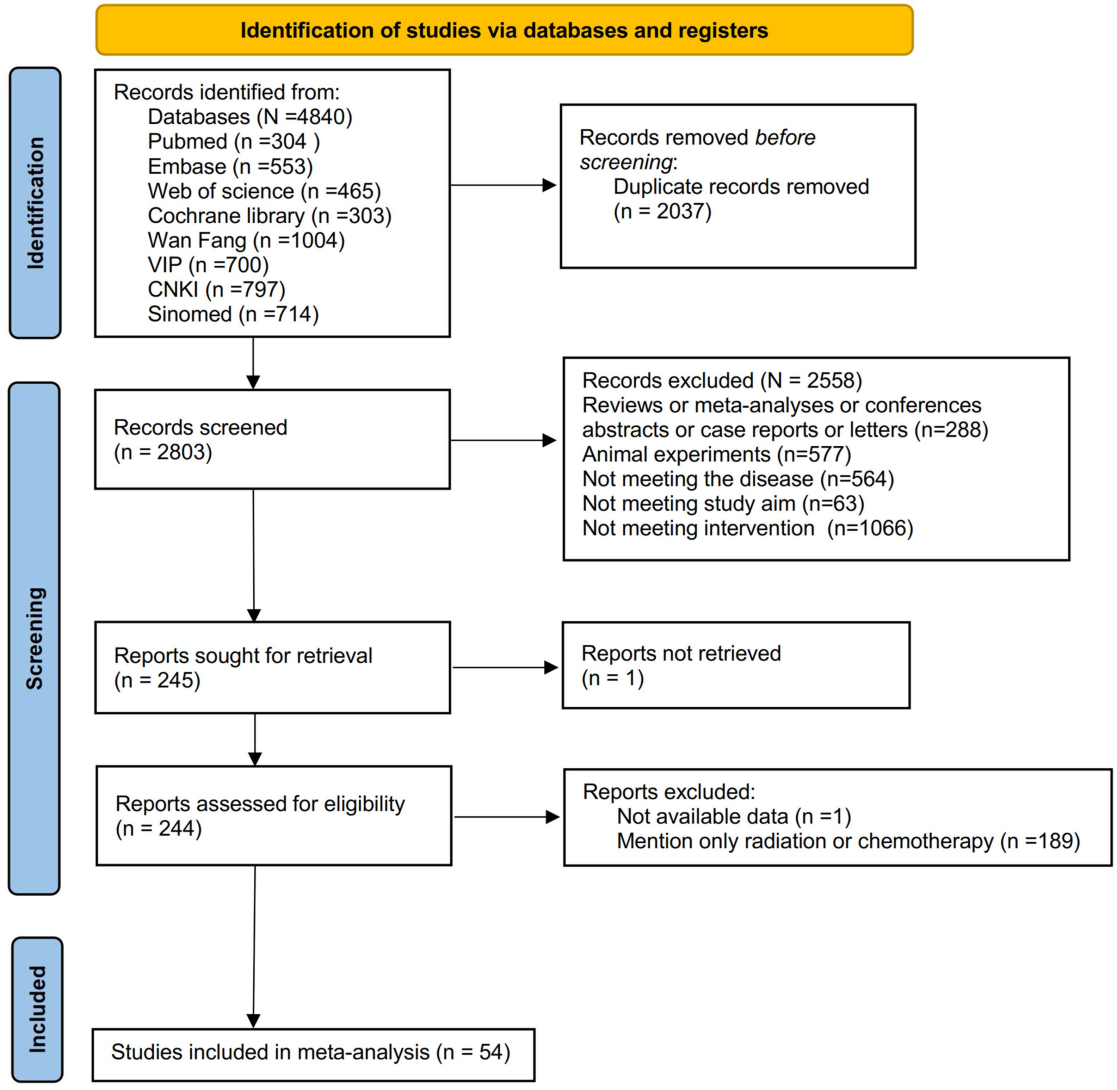
Study identification and selection flowchart.

4,840 articles were identified initially after a literature search. Following the removal of 2,037 duplicates, 2,558 articles were deleted after a review of titles and abstracts. Subsequently, the full texts of the remaining publications were assessed for eligibility. Ultimately, 54 studies (46–99) were eligible. The literature screening process is illustrated in Figure 1.

### 3.2 Basic characteristics of the included study

The 54 eligible studies (46–99) were all conducted in China and involved 4,201 patients. Among them, 2,113 patients in the experimental cohort received CMIs+CCRT, while 2,088 in the control cohort received only CCRT. Most patients had squamous cell carcinoma (SCC) or adenocarcinoma (AC). Two studies (74, 83) focused on adenosquamous carcinoma (ASCC), and another two studies (85, 87) included cases of undifferentiated carcinoma (UDC). The TNM staging of patients ranged from stage I to IV, and the intervention durations varied from 10 to 112 days. 13 types of CMIs were involved, including AD (11 RCTs), FFKS (11 RCTs), SM (4 RCTs), SF (1 RCT), KLT (3 RCTs), KA (4 RCTs), HQDT (1 RCT), SQFZ (1 RCT), XAP (3 RCTs), XYP (1 RCT), KSS (2 RCTs), LXX (5 RCTs), and YDZYR (7 RCTs). Detailed study characteristics are presented in Table 1 and Supplementary material 4.

**Table 1:** The characteristics of the included studies.

Study	Sample (I/C)	Gender (M/F)	Age (years) (I/C)	TNM clinical stage	Caner type (number of cases)	Intervention	Control	Duration	Outcomes
Zhao et al. (46)	34/31	45/20	18–71/40–72	III + IV	Unknown	KLT 100 mL + CCRT	CCRT (\ + CF 200 mg/m^2^ + 5-FU 500 mg/m^2^ + DDP 20 mg/m^2^)	21d × 3	①⑥⑧
Yu et al. (47)	53/53	Unknown	20–71	III + IV	SCC (92) + AC (14)	AD 50 mL + CCRT	CCRT (60-70Gy + CF 200 mg/m^2^ + 5-FU 500 mg/m^2^ + DDP 20 mg/m^2^)	20d × 2	①⑧
Li et al. (48)	37/37	Unknown	40–75	III + IV	SCC (71) + AC (3)	FFKS 30 mL + CCRT	CCRT (60-70Gy + 5-FU 500 mg/m^2^ + DDP 30 mg/m^2^)	21d	①⑧
Zhao et al. (49)	22/21	Unknown	49–75	III + IV	SCC (43)	AD 50 ml + CCRT	CCRT (60Gy + 5-FU 700 mg/m^2^ + DDP 52.5 mg/m^2^)	10d	①⑦⑧
He et al. (50)	38/38	Unknown	45–78	Unknown	SCC (76)	FFKS 15 mL + CCRT	CCRT (66-68Gy + PTX 150 mg/m^2^ + DDP 80 mg/m^2^)	25-28d	①⑥⑧
Yue et al. (51)	100/100	134/66	55/56	III + IV	Unknown	YDZYR 20-30 mL + CCRT	CCRT (60-66Gy + 5-FU 500 mg/m^2^ + DDP 15 mg/m^2^)	28d × 2	①⑦⑧
Pu (52)	47/47	48/46	53/55	III + IV	SCC (67) + AC (27)	SM 60 mL + CCRT	CCRT (60-66Gy + 5-FU 500 mg/m^2^ + DDP 20 mg/m^2^)	28d	①⑧
Pu(a) (53)	43/43	Unknown	63	III + IV	Unknown	FFKS 20 mL + CCRT	CCRT (60-70Gy + PTX 135 mg/m^2^ + DDP 80 mg/m^2^)	21d	①⑧
Sun (54)	40/40	52/28	42–75/40–75	IV	SCC (80)	FFKS 20 mL + CCRT	CCRT (60Gy + 5-FU 250 mg/m^2^ + DDP 10 mg/m^2^)	10d × (3–4)	①⑥
Wang (55)	31/31	Unknown	56	III + IV	SCC (54) +AC (6)	SF 100 ml + CCRT	CCRT (60Gy + 5-FU 500 mg/m^2^ + DDP 20 mg/m^2^)	5d × 3	①⑥
Yang et al. (56)	36/36	Unknown	60	III + IV	SCC (72)	FFKS 20 mL + CCRT	CCRT (64-68Gy + 5-FU 500 mg/m^2^ + DDP 20 mg/m^2^)	21d	①⑧
Zhao et al. (57)	31/31	43/19	43–69/40–71	Unknown	SCC (62)	AD 80 mL + CCRT	CCRT (66-70Gy + CF 150 mg/m^2^ + 5-FU 350 mg/m^2^ + DDP 35 mg/m^2^)	14d × 2	①⑦⑧
Zhao et al. (58)	32/30	43/19	Unknown	III + IV	SCC (59) + AC (3)	KSS 30 mL + CCRT	CCRT (60Gy + 5-FU 750 mg/m^2^ + DDP 75 mg/m^2^)	20d	①⑧
Lin (59)	31/31	Unknown	Unknown	III + IV	Unknown	AD 80 mL + CCRT	CCRT (66-70Gy + CF 150 mg/m^2^ + 5-FU 350 mg/m^2^ + DDP 35 mg/m^2^)	14d × 2	①⑧
Lu et al. (60)	29/29	43/15	36–74/38–73	III + IV	SCC (58)	YDZYR 30 mL + CCRT	CCRT (60-64Gy + L-OHP 85 mg/m^2^ + CF 250 mg/m^2^ + 5-FU 400-600 mg/m^2^)	28d	①⑥⑧
Shang et al. (61)	30/30	38/22	53.2 ± 10.1/54.8 ± 9.5	III + IV	SCC (60)	FFKS 20 mL + CCRT	CCRT (60Gy + PTX 100 mg/m^2^ + DDP 20 mg/m^2^)	10d	①⑥⑧
Zhong et al. (62)	30/30	35/25	52.9 ± 6.1/53.3 ± 5.6	III + IV	SCC (39) + AC (21)	LXX 500 mg + CCRT	CCRT (60-66Gy + 5-FU 500 mg/m^2^ + DDP 20 mg/m^2^)	28d × 2	①⑦⑧
Zhou et al. (63)	42/42	44/40	51/50	III + IV	SCC (58) + AC (26)	LXX 500 mg + CCRT	CCRT (60-66Gy + 5-FU 500 mg/m^2^ + DDP 20 mg/m^2^)	28d × 2	①⑦⑧
Chen et al. (64)	25/21	Unknown	55–75	III + IV	Unknown	SQFZ 250 mL + CCRT	CCRT (\ + PTX 135 mg/m^2^ + DDP 40 mg/m^2^)	14d	①
Cheng et al. (65)	34/33	51/16	55.7 ± 10.4/56.1 ± 9.8	III + IV	SCC (67)	KA 40 mL + CCRT	CCRT (40-50Gy + 5-FU 500 mg/m^2^ + DDP 20 mg/m^2^)	42d	①⑧
Liu et al. (66)	23/23	29/17	41–73/40–68	III	SCC (46)	YDZYR 30 mg + CCRT	CCRT (50.4Gy + PTX 45-60 mg/m^2^)	5d × 6	①
Luo (67)	36/36	Unknown	57.4 ± 5.6	III + IV	Unknown	KSS 30 mL + CCRT	CCRT (60Gy + 5-FU 750 mg/m^2^ + DDP 75 mg/m^2^)	20d	①
Chen (68)	44/44	Unknown	53.3 ± 4.1	III + IV	SCC (88)	SM 60 mL + CCRT	CCRT (60-66Gy + 5-FU 500 mg/m^2^ + DDP 20 mg/m^2^)	28d	①⑧
Liu (69)	38/38	Unknown	40–75	III + IV	SCC (72) + AC (4)	FFKS 20 mL + CCRT	CCRT (60-64Gy + 5-FU 500 mg/m^2^ + DDP 30 mg/m^2^)	50d	①
Liu et al. (70)	25/15	Unknown	Unknown	III + IV	Unknown	YDZYR 20-30 mL + CCRT	CCRT (60-64Gy + 5-FU 500 mg/m^2^ + DDP 15 mg/m^2^)	40d	⑦⑧
Lv et al. (71)	43/43	66/20	54.8 ± 8.2/55.3 ± 7.9	III + IV	SCC (86)	KA 40 mL + CCRT	CCRT (40-50Gy + 5-FU 500 mg/m^2^ + DDP 20 mg/m^2^)	42d	①②③④⑤⑧
Wang (72)	25/25	39/11	56 ± 5.4/55 ± 4.2	Unknown	Unknown	AD 80 ml + CCRT	CCRT (60Gy + CF 150 mg/m^2^ + 5-FU 350 mg/m^2^ + DDP 35 mg/m^2^)	14d × 2	①
Wu (73)	40/40	52/28	66 ± 8/68 ± 6	II + III	SCC (80)	XAP 60 mL + CCRT	CCRT (60-66Gy + DDP 35-40 mg/m^2^)	21d	①⑧
Cai et al. (74)	37/37	44/30	52.46 ± 7.25/54.12 ± 7.64	III + IV	SCC (58) + AC (5) + ASCC (11)	LXX 500 mg + CCRT	CCRT (60-70Gy + DDP 30 mg/m^2^)	5d × (8–10)	①⑧
Feng et al. (75)	46/46	61/31	49.53 ± 5.98/52.08 ± 6.23	IV	SCC (82) + AC (10)	AD 50 mL + CCRT	CCRT (50-60Gy + CF 300 mg/m^2^ + 5-FU 750 mg/m^2^ + DDP 40 mg/m^2^)	14d × 2	①②③④⑧
Jiang (76)	30/30	Unknown	52.1 ± 10.3	III + IV	SCC (60)	AD + CCRT	CCRT (60Gy + 5-FU 700 mg/m^2^ + DDP 52.5 mg/m^2^)	14d × 2	①⑥⑧
Liu et al. (77)	46/46	53/39	58.29 ± 4.06/59.33 ± 3.97	II + III + IV	Unknown	AD 50 mL + CCRT	CCRT (60-66Gy + CF 200 mg/m^2^ + 5-FU 500 mg/m^2^ + DDP 20 mg/m^2^)	20d × 3	①②③④⑥
Pan et al. (78)	41/41	53/29	58.6 ± 5.6/59.1 ± 5.5	III + IV	Unknown	AD 80 ml + CCRT	CCRT (66-70Gy + DOC 75 mg/m^2^ + DDP 20 mg/m^2^)	14d × 2	①②③④⑦⑧
Zhou et al. (79)	40/40	45/35	55.2 ± 15.5/54.8 ± 16.3	IV	SCC (71) + AC (9)	KA 40 ml + CCRT	CCRT (60Gy + 5-FU 500 mg/m^2^ + DDP 20 mg/m^2^)	5d × (6–7)	①③④⑤⑧
Huang et al. (80)	41/41	60/22	63.41 ± 7.82/62.85 ± 7.65	III + IV	SCC (77) + AC (5)	HQDT 250 mg + CCRT	CCRT (40-72Gy + 5-FU 500 mg/m^2^ + DDP 20 mg/m^2^)	(5–6) d × 12	①
Cheng et al. (81)	38/39	44/33	51–72/49–71	I + II + III +IV	SCC (77)	XAP 60 mL + CCRT	CCRT (60Gy + 5-FU 750 mg/m^2^ + DDP 75 mg/m^2^)	21d × 2	①⑧
Cui (82)	42/42	57/27	58.3 ± 4.7/59.6 ± 5.8	III + IV	SCC (76) + AC (8)	YDZYR 30 ml + CCRT	CCRT (60Gy + 5-FU 100 mg/m^2^ + DDP 20 mg/m^2^)	(18–27) d	①⑦
Han et al. (83)	59/59	92/26	72.15 ± 5.38/69.84 ± 5.97	Unknown	SCC (72) + AC (31) + ASCC (15)	FFKS 20 ml + CCRT	CCRT (40-50Gy + PTX 45 mg/m^2^ + DDP 75-80 mg/m^2^)	21d	①③④⑤⑦⑧
Xiu et al. (84)	16/16	17/15	55.98 ± 5.44/55.23 ± 6.32	III + IV	Unknown	AD 80 ml + CCRT	CCRT (66-70Gy + DOC 75 mg/m^2^ + DDP 20 mg/m^2^)	14d × 2	①②③④
Zhai (85)	30/30	37/23	49–79/47–75	III + IV	SCC (54) + AC (1) + UDC (5)	YDZYR 30 ml + CCRT	CCRT (60-64Gy + PTX 75 mg/m^2^ + NDP 25 mg/m^2^)	21d × 2	①⑥⑧
Chen (86)	52/52	67/37	64.2 ± 0.9/61.2 ± 0.8	III + IV	Unknown	AD 10 mL + CCRT	CCRT (50-70Gy + 5-FU 500 mg/m^2^ + DDP 30 mg/m^2^)	56d	①⑧
Lai et al. (87)	23/22	30/15	53.28 ± 8.26/52.97 ± 7.98	III + IV	SCC (3) + AC (39) + UDC (3)	YDZYR 20 ml + CCRT	CCRT (60Gy + DOC 75 mg/m^2^ + NDP 80 mg/m^2^)	30d	①③④⑤⑧
Zhang (88)	30/30	35/25	64.17 ± 7.40/64.30 ± 7.51	III + IV	SCC (60)	LXX 80 ml + CCRT	CCRT (59.4Gy + PTX 135 mg/m^2^ + DDP 20 mg/m^2^)	21d	①⑧
Dong et al. (89)	43/43	49/37	68.3 ± 6.2/67.7 ± 6.1	II + III	SCC (86)	KLT 200 mL + CCRT	CCRT (50-60Gy + S-1 40-60 mg, bid)	21d	①⑦⑧
Liu et al. (90)	60/60	92/28	72.11 ± 3.57/71.03 ± 4.67	III + IV	SCC (120)	SM 60 mL + CCRT	CCRT (45-56Gy + CAPE 1250 mg/m^2^)	(25–28) d	①⑧
Lu et al. (91)	40/40	52/28	71.35 ± 4.12/71.52 ± 3.69	II + III + IV	Unknown	FFKS 20 mL + CCRT	CCRT (≤60Gy + 5-FU 800 mg/m^2^ + DDP 20 mg/m^2^)	28d × (2–4)	①②③⑤⑥⑧
Chen (92)	60/60	81/39	61.26 ± 4.17/60.39 ± 4.26	III + IV	SCC (88) + AC (32)	XAP 60 mL + CCRT	CCRT (60Gy + 5-FU 750 mg/m^2^ + DDP 75 mg/m^2^)	28d × 4	①②③④⑧
Cheng et al. (93)	48/48	54/42	70.77 ± 6.86/71.25 ± 7.10	IV	SCC (96)	KA 60 mL + CCRT	CCRT (60Gy + S-1 60 mg, bid)	42d	①③④⑤⑧
Liu et al. (94)	35/35	59/11	66.74 ± 7.14/69.51 ± 9.18	Unknown	SCC (70)	XYP 500 mg + CCRT	CCRT (50-60Gy + PTX 50 mg/m^2^ + CBP AUC 2)	5d × (5–6)	⑧
Mao et al. (95)	34/34	48/20	51.33 ± 6.03/50.91 ± 5.91	III	SCC (68)	FFKS 15 mL + CCRT	CCRT (60Gy + 5-FU 1000 mg/m^2^ + DDP 75 mg/m^2^)	21d × 4	①⑧
An et al. (96)	48/48	51/45	59.70 ± 4.82/59.60 ± 4.45	III + IV	Unknown	FFKS 12 mL + CCRT	CCRT (60-66Gy + PTX 135-175 mg/m^2^ + NDP 80 mg/m^2^)	14d × 4	①③④⑧
Tian et al. (97)	30/30	34/26	68.14 ± 1.22/68.42 ± 1.32	II + III	Unknown	KLT 200 mL + CCRT	CCRT (50-60Gy + S-1 40-60 mg, bid)	21d	①⑦⑧
Wang et al. (98)	47/43	78/12	71.85 ± 9.44/73.47 ± 9.40	III + IV	SCC (90)	SM 100 mL + CCRT	CCRT (\ + S-1150 mg, bid)	5d/w	①⑧
Wang et al. (99)	58/58	77/39	55.01 ± 4.79/54.85 ± 4.76	III + IV	SCC (96) + AC (20)	LXX 400 mg + CCRT	CCRT (90Gy + PTX 175 mg/m^2^ + DDP 20 mg/m^2^)	21d × 3	①⑧

I, intervention group; C, control group; M, Male; F, female; SCC, squamous cell carcinoma; AC, adenocarcinoma; ASCC, adenosquamous cell carcinoma; UDC, undifferentiated carcinoma; KLT, kanglaite injection; AD, aidi injection; FFKS, fufangkushen injection; SM, shenmai injection; SF, shenfu injection; KA, kangai injection; HQDT, astragalus polysaccharides injection; SQFZ, shenqifuzheng injection; XAP, xiaoaiping injection; XYP, xiyanping injection; KSS, matrine injection; LXX, elemene injection; YDZYR, brucea javanica oil emulsion Injection; CCRT, concurrent chemoradiotherapy; CF, calcium folinate; 5-FU, 5-fluorouracil; DDP, cisplatin; PTX, paclitaxel; L-OHP, oxaliplatin; DOC, docetaxel; NDP, nedaplatin; S-1, tegafur, gimeracil, and oteracil potassium capsules; CAPE, capecitabine; CBP, carboplatin.

Outcomes: ① Clinical effectiveness rate; ② CD3+; ③CD4+; ④ CD8+; ⑤ CD4+/CD8+; ⑥ Performance status; ⑦ Survival rate (1 year); ⑧ Adverse reactions.

### 3.3 Methodological quality assessment of the included studies

**Figure 2:**
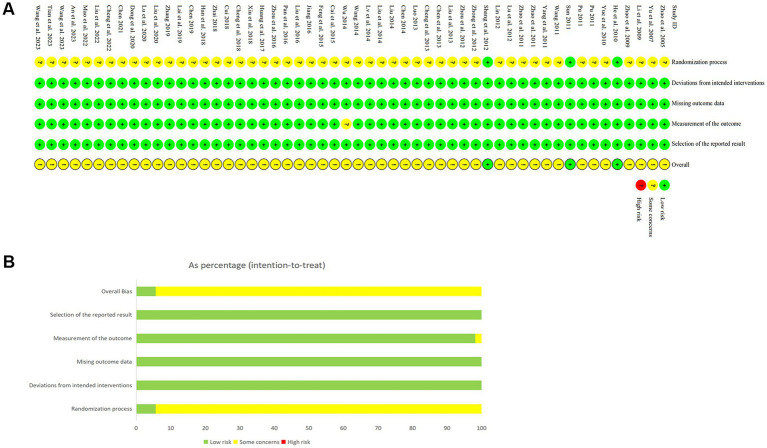
Risk of bias assessment. **(A)** Detailed assessment of risk of bias in included studies. **(B)** Summary of risk of bias in included studies.

The risk of bias assessment results are presented in Figure 2. With respect to bias arising from the randomization process, 51 studies were considered to have a potential risk owing to insufficient information regarding random sequence generation or the absence of allocation concealment, whereas 3 studies were assessed as being at low risk. In terms of outcome measurement, one RCT was identified as having a potential risk due to reporting only percentages without providing the absolute number of participants. All studies were judged to be at low risk of bias concerning deviations from intended interventions, missing outcome data, and selective outcome reporting. Overall, the included studies were determined to have a generally low risk of bias.

### 3.4 Network analysis results

#### 3.4.1 Network diagram

**Figure 3:**
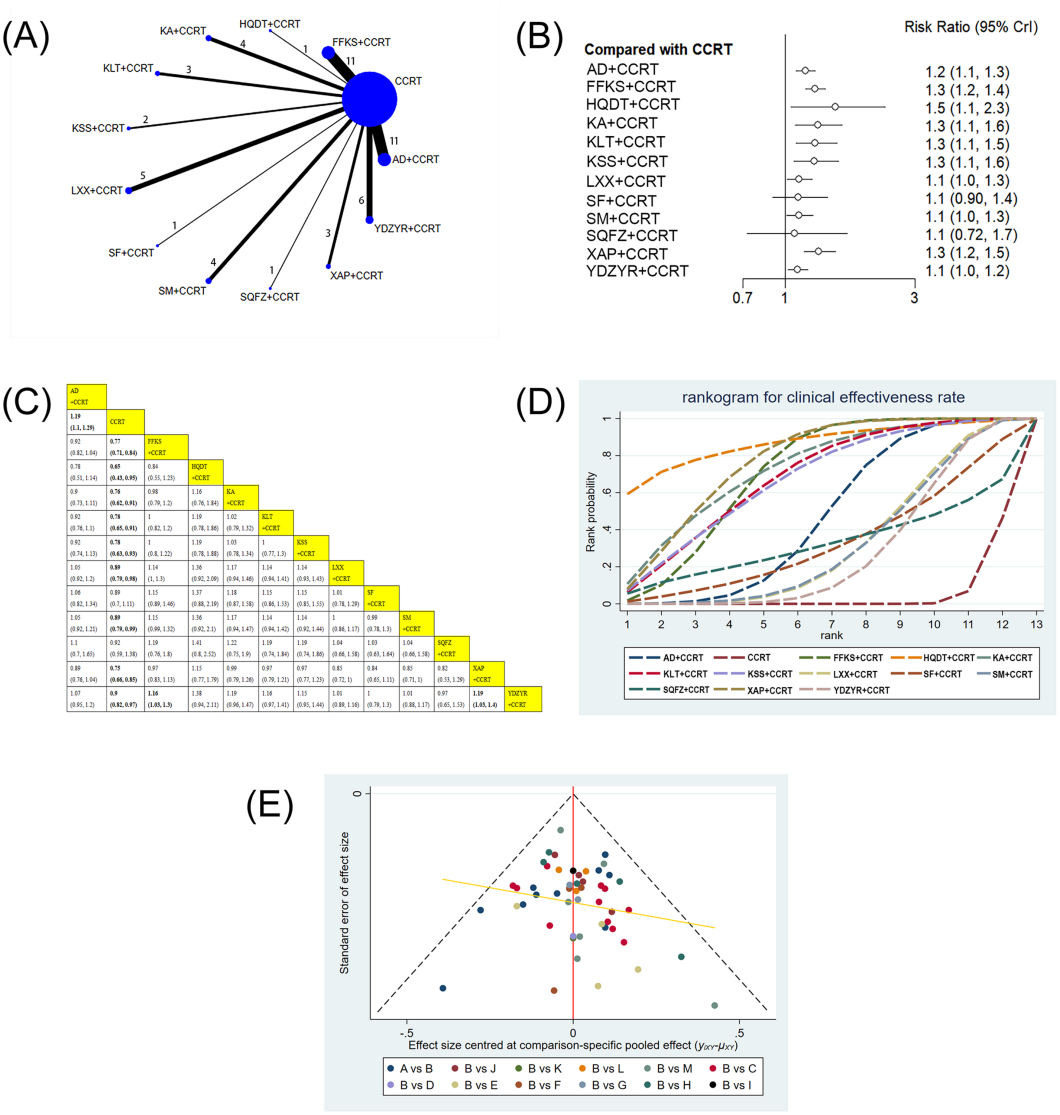
**(A)** Network graphs of clinical effectiveness rate. **(B)** Forest plot of clinical effectiveness rate. **(C)** League table of clinical effectiveness rate. **(D)** Cumulative probability line chart of clinical effectiveness rate. **(E)** Funnel plot of clinical effectiveness rate: A: AD+CCRT; B: CCRT; C: FFKS+CCRT; D: HQDT+CCRT; E: KA + CCRT; F: KLT + CCRT; G: KKS + CCRT; H: LXX + CCRT; I: SF + CCRT; J: SM + CCRT; K: SQFZ+CCRT; L: XAP + CCRT; M: YDZYR+CCRT.

**Figure 4:**
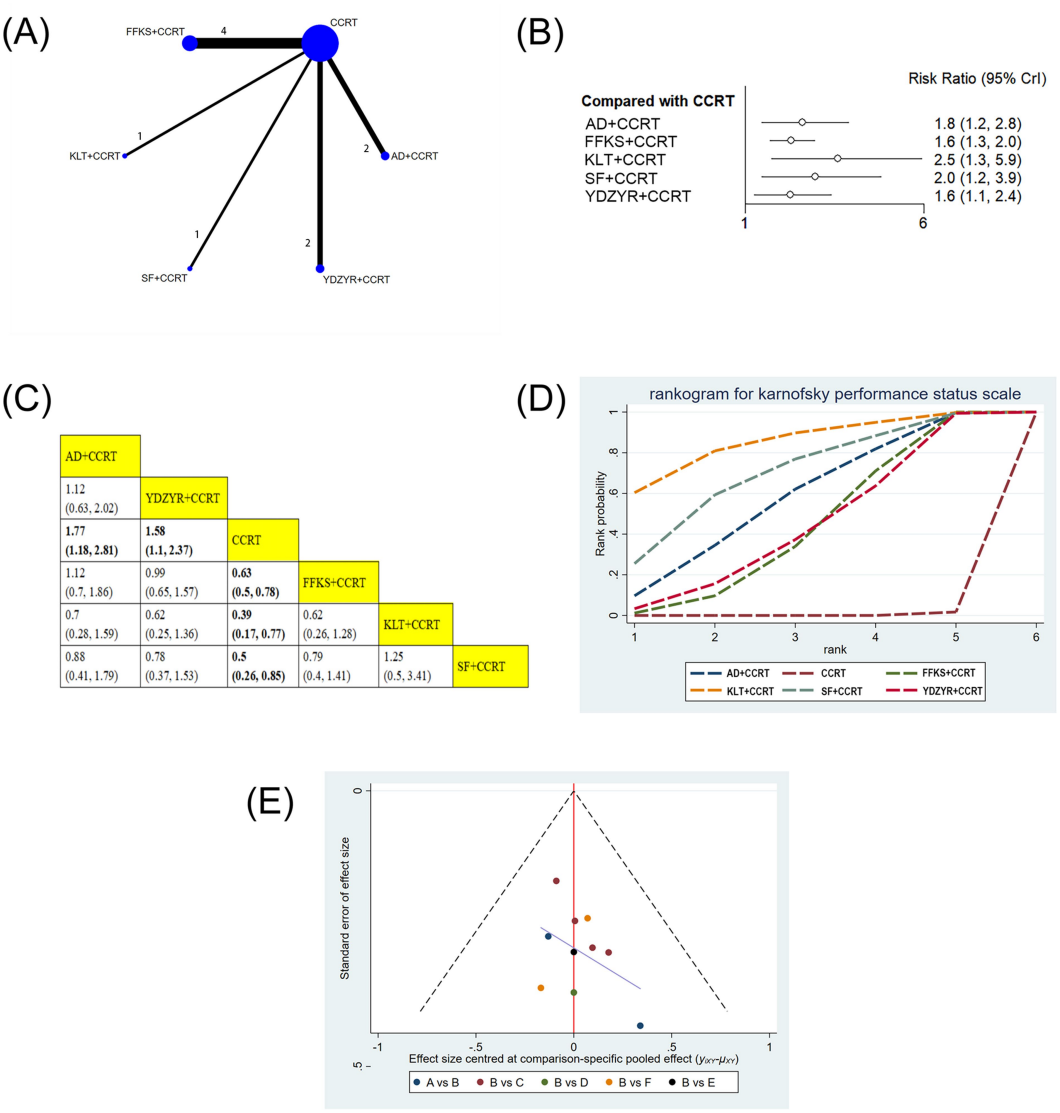
**(A)** Network graphs of performance status. **(B)** Forest plot of performance status. **(C)** League table of performance status. **(D)** Cumulative probability line chart of performance status. **(E)** Funnel plot of performance status: A: AD+CCRT; B: CCRT; C: FFKS+CCRT; D: KLT + CCRT; E: SF + CCRT; F: YDZYR+CCRT.

**Figure 5:**
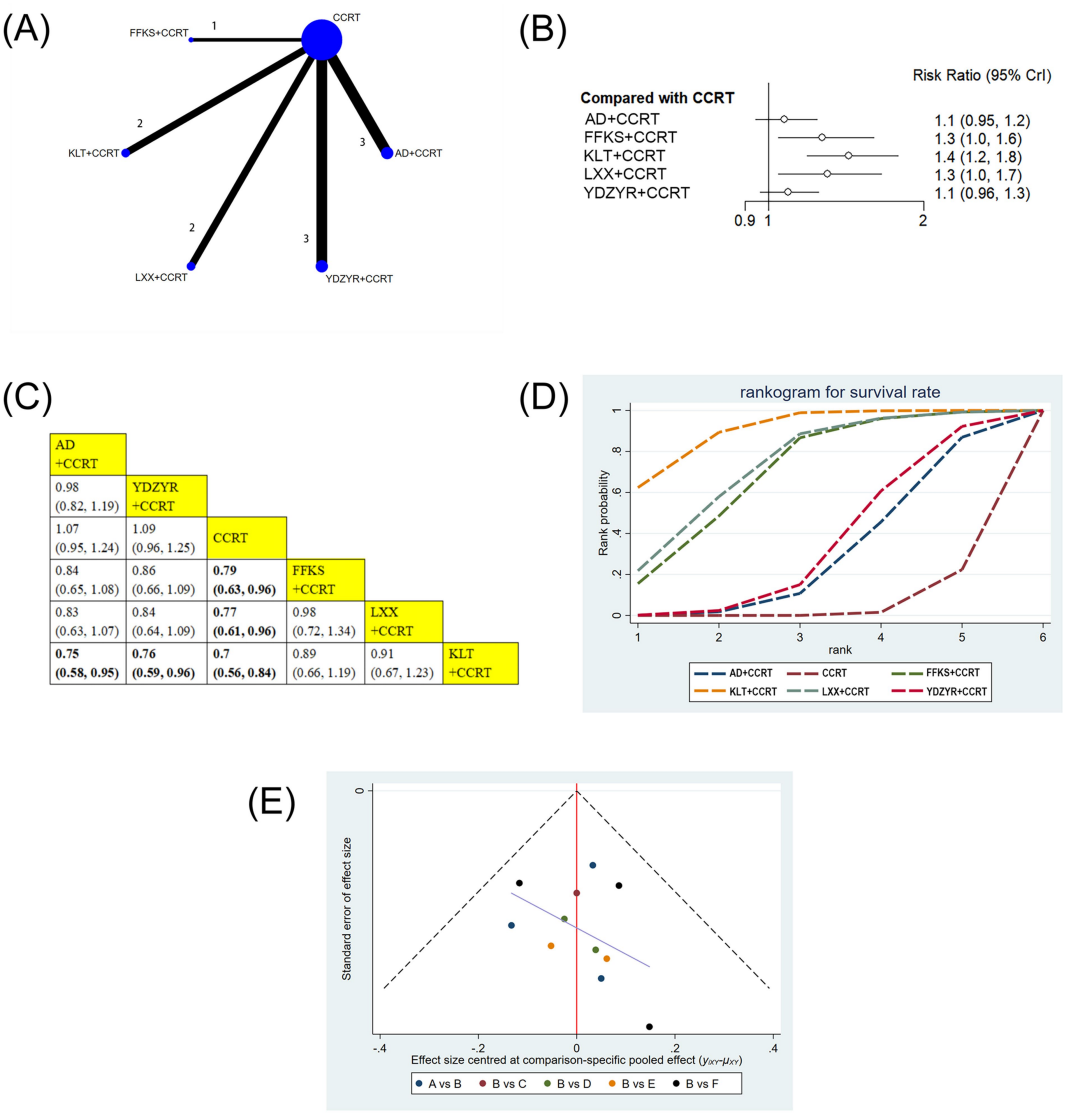
**(A)** Network graphs of survival rate. **(B)** Forest plot of survival rate. **(C)** League table of survival rate. **(D)** Cumulative probability line chart of survival rate. **(E)** Funnel plot of survival rate: A: AD+CCRT; B: CCRT; C: FFKS+CCRT; D: KLT + CCRT; E: LXX + CCRT; F: YDZYR+CCRT.

**Figure 6:**
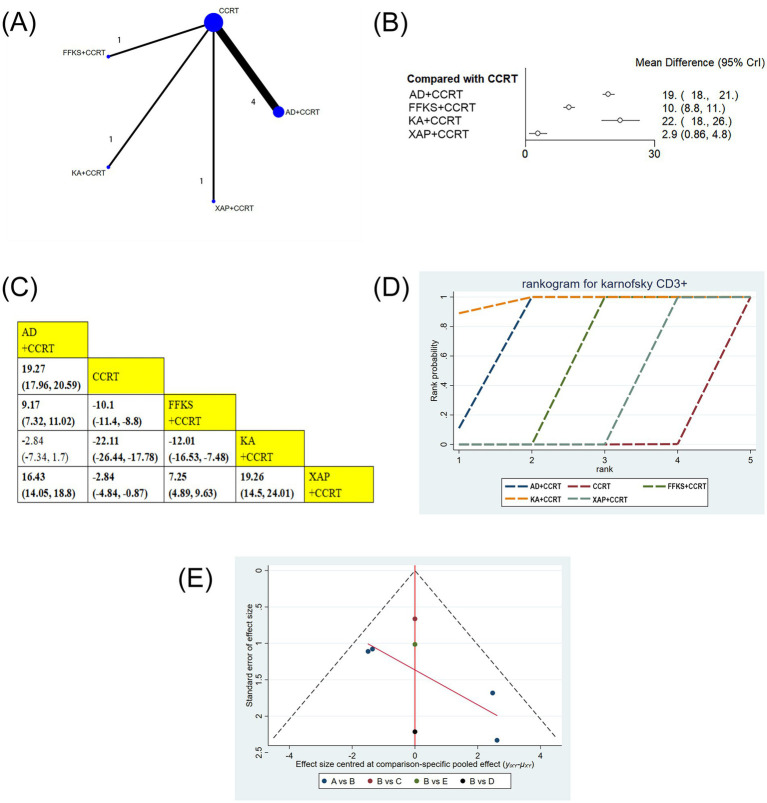
**(A)** Network graphs of CD3+. **(B)** Forest plot of CD3+. **(C)** League table of CD3+. **(D)** Cumulative probability line chart of CD3+. **(E)** Funnel plot of CD3+: A: AD+CCRT; B: CCRT; C: FFKS+CCRT; D: KA + CCRT; E: XAP + CCRT.

**Figure 7:**
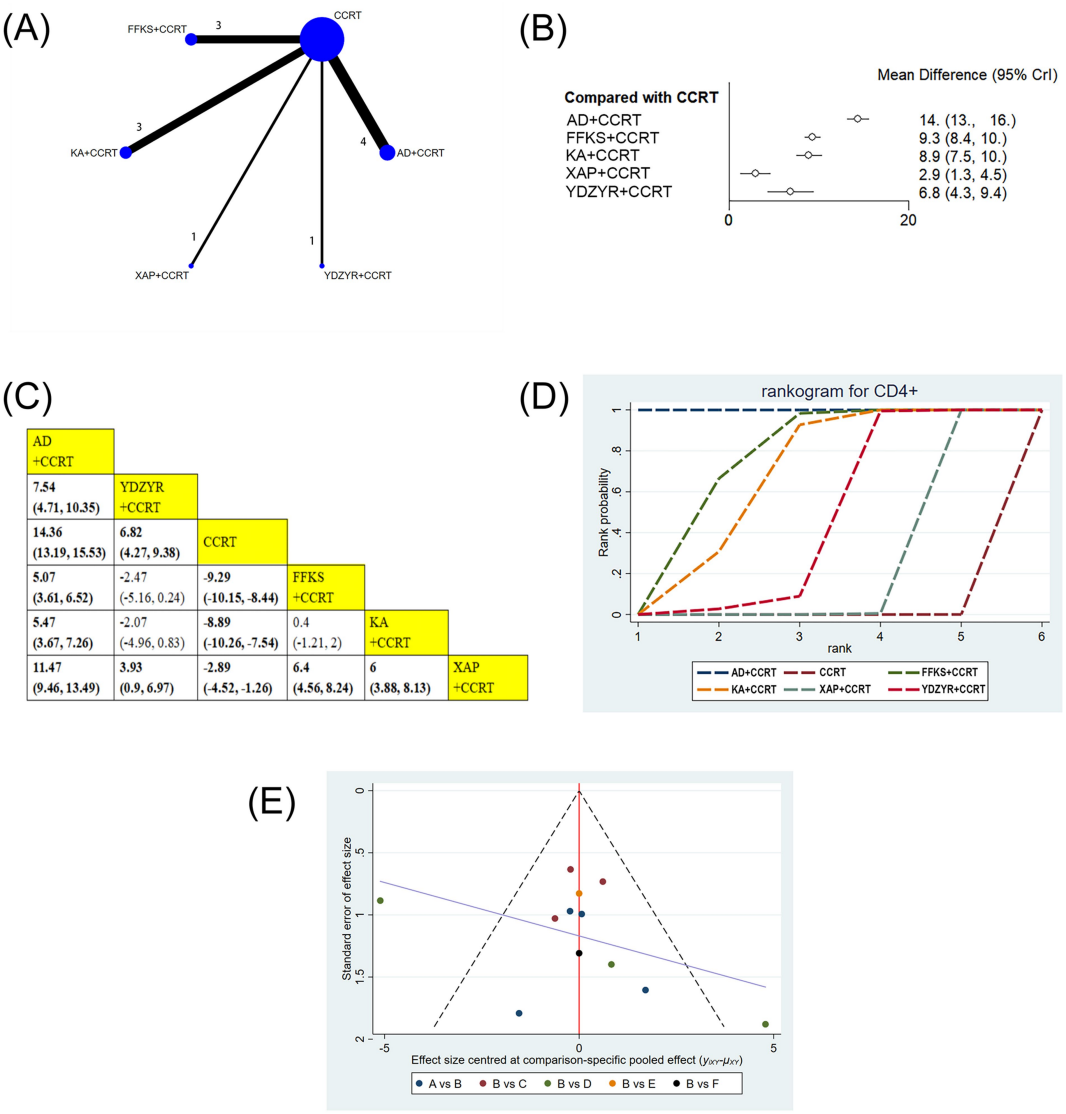
**(A)** Network graphs of CD4+. **(B)** Forest plot of CD4+. **(C)** League table of CD4+. **(D)** Cumulative probability line chart of CD4+. **(E)** Funnel plot of CD4+: A: AD+CCRT; B: CCRT; C: FFKS+CCRT; D: KA + CCRT; E: XAP + CCRT; F: YDZYR+CCRT.

**Figure 8:**
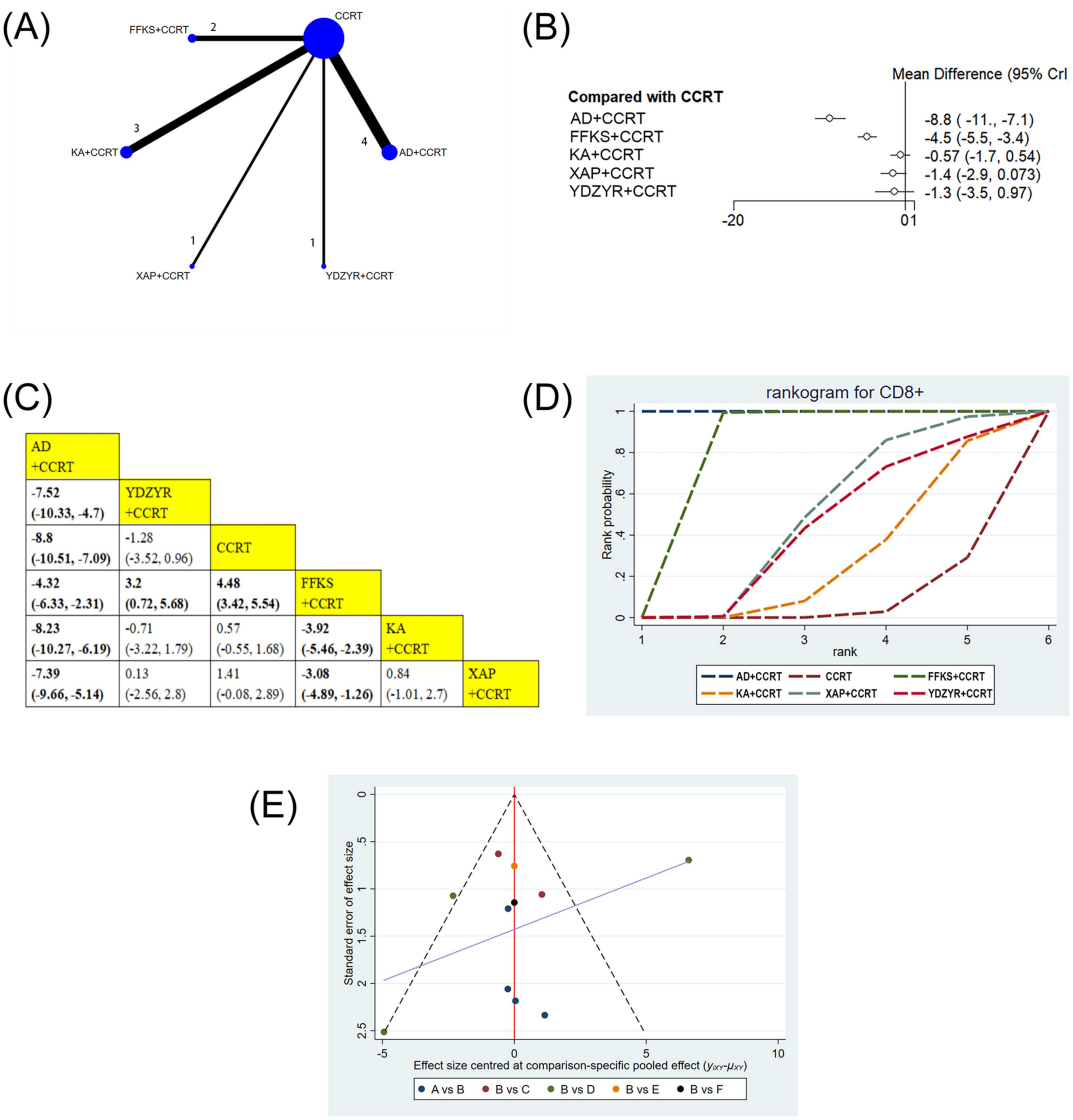
**(A)** Network graphs of CD8+. **(B)**. Forest plot of CD8+. **(C)** League table of CD8+. **(D)** Cumulative probability line chart of CD8+. **(E)** Funnel plot of CD8+: A: AD+CCRT; B: CCRT; C: FFKS+CCRT; D: KA + CCRT; E: XAP + CCRT; F: YDZYR+CCRT.

**Figure 9:**
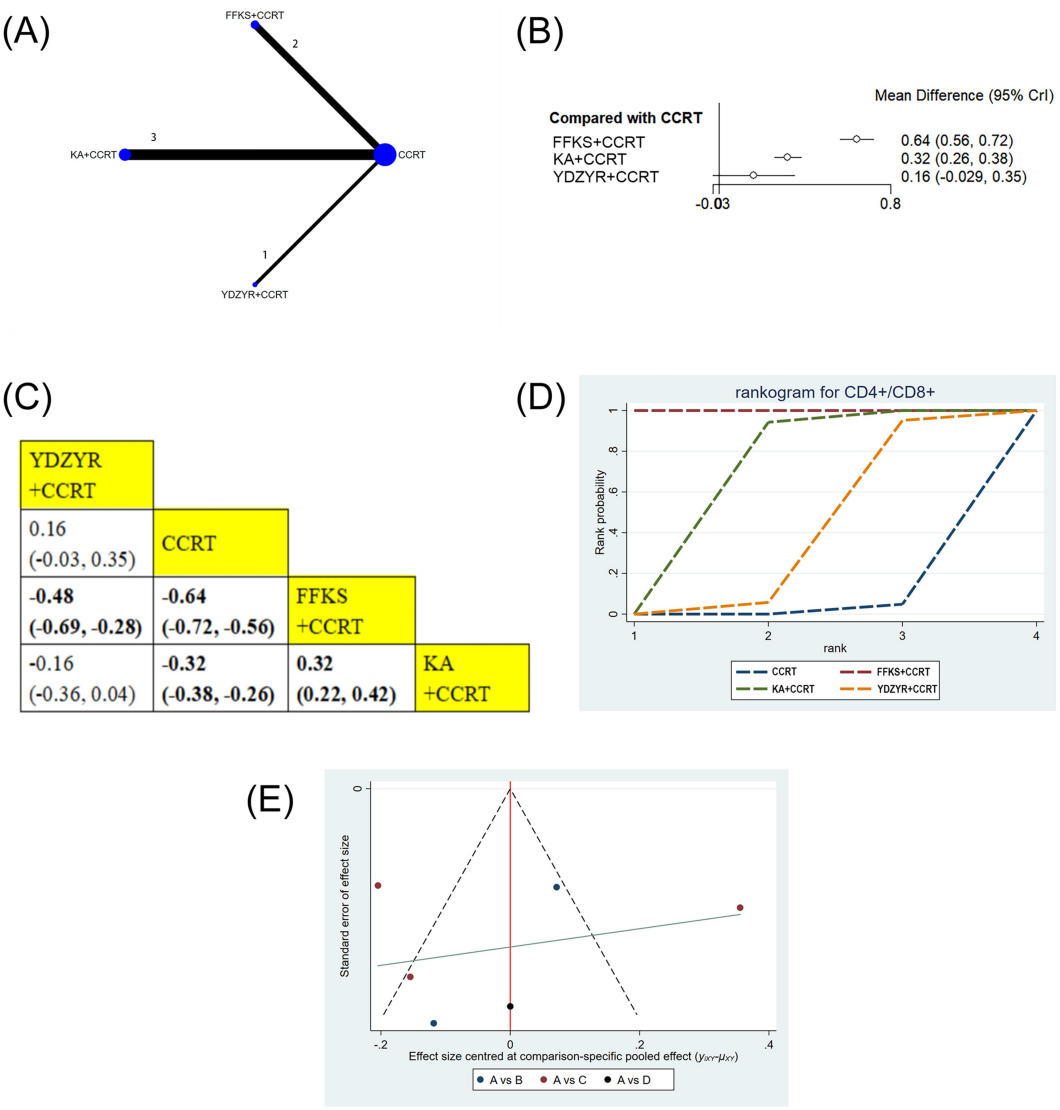
**(A)** Network graphs of CD4+/CD8+, **(B)** Forest plot of CD4+/CD8+. **(C)** League table of CD4+/CD8+. **(D)** Cumulative probability line chart of CD4+/CD8+. **(E)** Funnel plot of CD4+/CD8+: A: CCRT; B: FFKS+CCRT; C: KA + CCRT; D: YDZYR+CCRT.

The 54 included studies encompassed 13 distinct CMIs: AD, FFKS, SM, SF, KLT, KA, HQDT, SQFZ, XAP, XYP, KSS, LXX, and YDZYR. The network structure of these CMIs is shown in Figures 3A–9A. The line thickness is proportional to the number of studies comparing pairs of interventions. The circle diameter is proportional to participant number in each intervention.

#### 3.4.2 Clinical effectiveness rate

52 studies involving 4,091 patients reported the clinical effectiveness rate. Compared with CCRT alone, AD+CCRT (RR = 1.19, 95% CI: 1.10–1.29), FFKS+CCRT (RR = 0.77, 95% CI: 0.71–0.84), HQDT+CCRT (RR = 0.65, 95% CI: 0.43–0.95), KA + CCRT (RR = 0.76, 95% CI: 0.62–0.91), KLT + CCRT (RR = 0.78, 95% CI: 0.65–0.91), KSS + CCRT (RR = 0.78, 95% CI: 0.63–0.93), LXX + CCRT (RR = 0.89, 95% CI: 0.79–0.98), SM + CCRT (RR = 0.89, 95% CI: 0.79–0.99), XAP + CCRT (RR = 0.75, 95% CI: 0.66–0.85), and YDZYR+CCRT (RR = 0.90, 95% CI: 0.82–0.97) were all associated with significantly higher clinical effectiveness. Furthermore, FFKS+CCRT (RR = 1.16, 95% CI: 1.03–1.30) and XAP + CCRT (RR = 1.19, 95% CI: 1.03–1.40) showed significantly higher effectiveness than YDZYR+CCRT. No other pairwise comparisons demonstrated significant differences (Figures 3B,C). According to cumulative probability rankings, HQDT+CCRT (SUCRA = 86.7%), XAP + CCRT (SUCRA = 77.0%), and KA + CCRT (SUCRA = 73.0%) ranked highest for clinical effectiveness (Figure 3D and Table 2).

**Table 2:** Summary of SUCRA.

SUCRA	Clinical effectiveness rate	Performance status	Survival rate	CD3+	CD4+	CD8+	CD4+/CD8+
AD + CCRT	0.46679792	0.575698	0.29047	0.7775637	0.996998	0.998985	—
CCRT	0.04460542	0.003406	0.047849	0.0006525	0.000054	0.064263	0.01615667
FFKS + CCRT	0.70849208	0.43173	0.692006	0.50001	0.729628	0.798763	0.99999667
HQDT + CCRT	0.86669292	—	—	—	—	—	—
KA + CCRT	0.72962542	—	—	0.9724162	0.64698	0.263232	0.64748833
KLT + CCRT	0.68458	0.851393	0.900915	—	—	—	—
KSS + CCRT	0.67220917	—	—	—	—	—	—
LXX + CCRT	0.31749208	—	0.727619	—	—	—	—
SF + CCRT	0.32967625	0.699153	—	—	—	—	—
SM + CCRT	0.31427458	—	—	—	—	—	—
SQFZ+CCRT	0.32368333	—	—	—	—	—	—
XAP + CCRT	0.76954917	—	—	0.2493575	0.201022	0.46413	—
YDZYR+CCRT	0.27232167	0.43862	0.341141	—	0.422348	0.409623	0.33635833

#### 3.4.3 Performance status

10 studies involving 693 patients reported performance status. Compared with CCRT alone, AD+CCRT (RR = 1.77, 95% CI: 1.18–2.81), YDZYR+CCRT (RR = 1.58, 95% CI: 1.10–2.37), FFKS+CCRT (RR = 0.63, 95% CI: 0.50–0.78), KLT + CCRT (RR = 0.39, 95% CI: 0.17–0.77), and SF + CCRT (RR = 0.50, 95% CI: 0.26–0.85) significantly improved KPS. No significant differences were observed for other pairwise comparisons (Figures 4B,C). Based on cumulative probability results, KLT + CCRT (SUCRA = 85.1%), SF + CCRT (SUCRA = 69.9%), and AD+CCRT (SUCRA = 57.6%) were ranked as the top three regimens for improving performance status (Figure 4D and Table 2).

#### 3.4.4 Survival rate

11 studies involving 919 patients reported the one-year survival rate. The one-year survival rates for FFKS+CCRT (RR = 0.79, 95% CI: 0.63–0.96), LXX + CCRT (RR = 0.77, 95% CI: 0.61–0.96), and KLT + CCRT (RR = 0.70, 95% CI: 0.56–0.84) were notably higher in comparison to CCRT alone, with statistical significance. Furthermore, the one-year survival rate for KLT + CCRT was significantly higher than that for AD+CCRT (RR = 0.75, 95% CI: 0.58–0.95) and YDZYR+CCRT (RR = 0.76, 95% CI: 0.59–0.96). No significant differences were observed for other pairwise interventions (Figures 5B,C). Based on cumulative probability results, KLT + CCRT (SUCRA = 90.1%), LXX + CCRT (SUCRA = 72.8%), and FFKS+CCRT (SUCRA = 69.2%) were the top three effective schemes for increasing the one-year survival rate (Figure 5D and Table 2).

#### 3.4.5 CD3+

Seven studies involving 584 patients reported CD3 + levels. Compared with CCRT alone, AD+CCRT (MD = 19.27, 95% CI: 17.96–20.59), FFKS+CCRT (MD = −10.10, 95% CI: −11.40 to −8.80), KA + CCRT (MD = −22.11, 95% CI: −26.44 to −17.70), and XAP + CCRT (MD = −2.84, 95% CI: −4.84 to −0.87) significantly increased CD3 + levels. AD+CCRT outperformed FFKS+CCRT (MD = 9.17, 95% CI: 7.32–11.02) and XAP + CCRT (MD = 16.43, 95% CI: 14.05–18.80), while KA + CCRT showed higher levels than FFKS+CCRT (MD = −12.01, 95% CI: −16.53 to −7.48) and XAP + CCRT (MD = 19.26, 95% CI: 14.50–24.01). FFKS+CCRT also exceeded XAP + CCRT (MD = 7.25, 95% CI: 4.89–9.63). Statistically significant differences did not exist across other paired interventions (Figures 6B,C). Cumulative probability results revealed that KA + CCRT (SUCRA = 97.2%), AD+CCRT (SUCRA = 77.8%), and FFKS+CCRT (SUCRA = 50.0%) were the top three effective strategies for enhancing CD3 + levels (Figure 6D and Table 2).

#### 3.4.6 CD4+

12 studies involving 1,019 patients reported on CD4 + levels. The results showed that AD+CCRT (MD = 14.36, 95% CI: 13.19–15.53), YDZYR+CCRT (MD = 6.82, 95% CI: 4.27–9.38), FFKS+CCRT (MD = −9.29, 95% CI: −10.15 to −8.44), KA + CCRT (MD = −8.89, 95% CI: −10.26 to −7.54), and XAP + CCRT (MD = −2.89, 95% CI: −4.52 to −1.26) significantly elevated CD4 + levels compared to CCRT alone, with statistical significance. Additionally, AD+CCRT was significantly more effective than YDZYR+CCRT (MD = 7.54, 95% CI: 4.71–10.35), FFKS+CCRT (MD = 5.07, 95% CI: 3.61–6.52), KA + CCRT (MD = 5.47, 95% CI: 3.67–7.26), and XAP + CCRT (MD = 11.47, 95% CI: 9.46–13.49). CD4 + levels with YDZYR+CCRT (MD = 3.93, 95% CI: 0.9–6.97), FFKS+CCRT (MD = 6.40, 95% CI: 4.56–8.24), and KA + CCRT (MD = 6.00, 95% CI: 3.88–8.13) were all significantly higher than those with XAP + CCRT. Statistically significant differences were not noted between other pairwise treatments(Figures 7B,C). Cumulative probability results demonstrated that AD+CCRT (SUCRA = 99.9%), FFKS+CCRT (SUCRA = 73.0%), and KA + CCRT (SUCRA = 64.7%) were the three most effective measures for enhancing CD4 + levels (Figure 7D and Table 2).

#### 3.4.7 CD8+

11 studies (939 patients) reported CD8 + levels. AD+CCRT (MD = −8.80, 95% CI: −10.51 to −7.09) and FFKS+CCRT (MD = 4.48, 95% CI: 3.42–5.54) showed significant increases versus CCRT alone. AD+CCRT outperformed YDZYR+CCRT (MD = −7.52, 95% CI: −10.33 to −4.70), FFKS+CCRT (MD = −4.32, 95% CI: −6.33 to −2.31), KA + CCRT (MD = −8.23, 95% CI: −10.27 to −6.19), and XAP + CCRT (MD = −7.39, 95% CI: −9.66 to −5.14). FFKS+CCRT surpassed YDZYR+CCRT (MD = 3.20, 95% CI: 0.72–5.68), KA + CCRT (MD = −3.92, 95% CI: −5.46 to −2.39), and XAP + CCRT (MD = −3.08, 95% CI: −4.89 to −1.26). No other significant differences were noted (Figures 8B,C). SUCRA rankings were AD+CCRT (SUCRA = 99.9%), FFKS+CCRT (SUCRA = 79.9%), XAP + CCRT (SUCRA = 46.4%) (Figure 8D and Table 2).

#### 3.4.8 CD4+/CD8+

Six studies (505 patients) reported the CD4+/CD8 + ratio. FFKS+CCRT (MD = −0.64, 95% CI: −0.72 to −0.56) and KA + CCRT (MD = −0.32, 95% CI: −0.38 to −0.26) significantly increased the ratio versus CCRT alone. FFKS+CCRT was superior to YDZYR+CCRT (MD = −0.48, 95% CI: −0.69 to −0.28) and KA + CCRT (MD = 0.32, 95% CI: 0.22–0.42). Other comparisons were nonsignificant (Figures 9B,C). SUCRA rankings were FFKS+CCRT (SUCRA = 99.9%), KA + CCRT (SUCRA = 64.7%), YDZYR+CCRT (SUCRA = 33.6%) (Figure 9D and Table 2).

### 3.5 Cluster analysis

**Figure 10:**
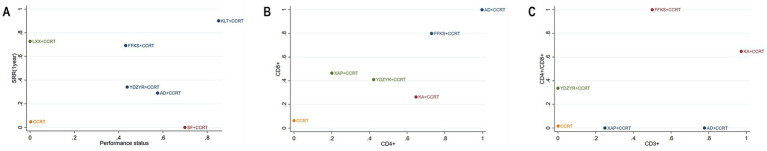
cluster plot. **(A)** Survival rate and Performance status; **(B)** CD8 + and CD4+; **(C)** CD4+/CD8 + and CD3+.

To determine the best treatment for EC based on key outcome markers, cluster analysis was performed. Two-dimensional results indicated AD+CCRT as the preferred treatment for improving CD4 + and CD8 + cell counts. For enhancing performance status and improving one-year survival rates, KLT + CCRT was identified as the most optimal approach. KA + CCRT, positioned furthest from the origin, demonstrated the greatest effect for improving CD3+. The combination of FFKS and radiochemotherapy was the most effective in enhancing CD4+/CD8 + (Figure 10).

### 3.6 AEs

43 studies reported AEs. It should be noted that all AEs in this study were descriptively summarized, without statistical comparisons between groups; therefore, the results are intended for clinical reference only and should be interpreted with caution. Reported AEs included hematotoxicity and bone marrow suppression, leukopenia, thrombocytopenia, anemia, neutropenia, gastrointestinal reactions, nausea and vomiting, anorexia, fatigue, radiation esophagitis, radiation pneumonia, abnormal liver and kidney function, renal impairment, hepatotoxicity, peripheral neuropathy, alopecia, fever, infection, esophageal or oral mucosal reactions, cutaneous allergic reactions, radiation enteritis, radiation cystitis, hypoproteinemia, and aspiration pneumonia. Specifically, hematotoxicity and bone marrow suppression were more frequently observed in the YDZYR+CCRT group (77.29%); leukopenia and thrombocytopenia were more common in the SM + CCRT group, with incidences of 31.82 and 30.81%, respectively; gastrointestinal reactions occurred at a relatively high frequency in the YDZYR+CCRT group (49.28%); nausea and vomiting were more frequent in the XAP + CCRT group (15.33%); anorexia, fatigue, and radiation pneumonia were more common in the KSS + CCRT group, with incidences of 15.63, 12.50, and 28.13%, respectively; radiation esophagitis occurred most frequently in the XYP + CCRT group (82.86%); abnormal liver and kidney function was more frequent in the LXX + CCRT group (9.64%); alopecia and peripheral neuropathy were also observed in the YDZYR+CCRT group, with incidences of 4.35 and 3.38%, respectively; fever and esophageal or oral mucosal reactions were more common in the AD+CCRT group, with incidences of 2.86 and 11.79%, respectively. Additionally, anemia (15.33%), neutropenia (5.00%), infection (1.07%), renal impairment (2.83%), cutaneous allergic reactions (12.26%), radiation enteritis (8.03%), radiation cystitis (9.49%), hypoproteinemia (14.65%), and aspiration pneumonia (1.21%) were each reported in a single study of CMIs. The results are summarized in Table 3. Overall, descriptive data indicate variation in the incidence of AEs across treatment groups. Notably, AD+CCRT and FFKS+CCRT demonstrated comparatively lower incidences of the most common AEs, hematotoxicity and bone marrow suppression, nausea and vomiting, and radiation esophagitis, suggesting a potentially more favorable safety profile. Importantly, none of the CMIs were associated with severe AEs. Future research employing well-designed prospective studies is warranted to further validate safety differences among CMIs and elucidate the underlying mechanisms (Table 3).

**Table 3:** Summary of adverse reactions.

Intervention	AD + CCRT	FFKS + CCRT	KA + CCRT	KLT + CCRT	KSS + CCRT	LXX + CCRT	SM + CCRT	XAP + CCRT	XYP + CCRT	YDZYR + CCRT	SQFZ + CCRT
Samplesize	280	365	165	106	32	197	198	137	35	207	46
Hematotoxicity and bone marrow suppression	37(13.21%)	38(10.41%)	32(19.39%)	44(41.51%)	3(9.38%)	38(19.29%)	28(14.14%)	12(8.76%)	—	160(77.29%)	25(54.35%)
Leukopenia	34(12.14%)	70(19.18%)	8(4.85%)	—	—	42(21.32%)	63(31.82%)	10(7.30%)	—	—	—
Thrombocytopenia	4(1.43%)	20(5.48%)	—	—	—	37(18.78%)	61(30.81%)	19(13.87%)	—	—	—
Anemia	—	—	—	—	—	—	—	21(15.33%)	—	—	—
Neutropenia	14(5.00%)	—	—	—	—	—	—	—	—	—	—
Gastrointestinal reactions	73(26.07%)	88(24.11%)	32(19.39%)	10(9.43%)	—	55(27.92%)	63(31.82%)	—	—	102(49.28%)	25(54.35%)
Nausea and vomiting	34(12.14%)	5(1.37%)	4(2.42%)	—	2(6.25%)	12(6.09%)	—	21(15.33%)	—	1(0.48%)	—
Anorexia	4(1.43%)	14(3.84%)	—	—	5(15.63%)	—	—	—	—	—	—
Fatigue	14(5.00%)	—	—	—	4(12.50%)	—	—	—	—	—	—
Radiation esophagitis	74(26.43%)	26(7.12%)	44(26.67%)	—	22(68.75%)	105(53.30%)	90(45.45%)	33(24.09%)	29(82.86%)	89(43.00%)	25(54.35%)
Radiation pneumonia	7(2.50%)	22(6.03%)	—	5(4.72%)	9(28.13%)	41(20.81%)	28(14.14%)	4(2.92%)	8(22.86%)	—	25(54.35%)
Abnormal liver and kidney function	5(1.79%)	3(0.82)	—	—	—	19(9.64%)	—	—	—	—	—
Renal impairment	—	—	—	3(2.83%)	—	—	—	—	—	—	—
Liver toxicity	—	—	—	7(6.60%)	—	13(6.60%)	—	—	—	—	—
Peripheral neuropathy	2(0.71%)	—	—	—	—	—	—	—	—	7(3.38%)	—
Alopecia	—	—	1(0.61%)	—	—	—	—	—	—	9(4.35%)	—
Fever	8(2.86%)	—	—	—	—	—	—	—	—	2(0.97%)	—
Infection	3(1.07%)	—	—	—	—	—	—	—	—	—	—
Esophageal or oral mucosal reactions	33(11.79%)	—	—	—	—	—	—	—	—	7(3.38%)	—
Skin allergy	—	—	—	13(12.26%)	—	—	—	—	—	—	—
Radiation enteritis	—	—	—	—	—	—	—	11(8.03%)	—	—	—
Radiation cystitis	—	—	—	—	—	—	—	13(9.49%)	—	—	—
Hypoproteinemia	—	—	—	—	—	—	29(14.65%)	—	—	—	—
Aspiration pneumonia	—	—	2(1.21%)	—	—	—	—	—	—	—	—

### 3.7 Consistency analysis, convergence diagnostics, and heterogeneity assessment

The consistency of the results was assessed by comparing the DIC values between the consistency and inconsistency models. For all outcome measures, the DIC differences were less than 5, indicating a high degree of concordance between the models; detailed results are provided in Supplementary material 5. Convergence diagnostics demonstrated that, following iterative computation, all outcome parameters steadily approached a PSRF of 1, suggesting that the results are robust and reliable (Supplementary material 6). Heterogeneity analysis revealed low heterogeneity for clinical effectiveness rate, performance status, and survival rate, whereas CD3^+^ exhibited moderate heterogeneity. In contrast, high heterogeneity was observed for CD4+, CD8+, and CD4+/CD8+. Clinically, this heterogeneity may be attributable to variations in patients’ baseline immune status, differences in treatment protocols, or inconsistencies in assay methodologies. Detailed findings are presented in Supplementary material 7.

### 3.8 Publication bias

Funnel plots and Egger’s test were employed to evaluate publication bias for all outcome indicators. The results of Egger’s test are detailed in Supplementary material 8. As illustrated in Figures 3E–7E, the funnel plots for clinical effectiveness rate, performance status, one-year survival rate, CD3+, and CD4 + appeared visually symmetrical, and Egger’s test revealed no significant differences (*p* > 0.05), indicating the absence of publication bias among these studies. In contrast, although Egger’s test for CD8 + and CD4+/CD8 + did not demonstrate significant differences (*p* > 0.05), the funnel plots were not fully symmetrical, suggesting the potential presence of some publication bias (Figures 8E, 9E).

### 3.9 CINeMA evidence evaluation

The quality of evidence for seven outcome indicators was assessed using the CINeMA framework. All evidence was classified as either “low” or “moderate.” For clinical effectiveness rate, most comparisons were rated “moderate,” with only CCRT versus SF + CCRT and CCRT versus SQFZ+CCRT downgraded to “low” due to severe imprecision. Evidence for performance status was primarily rated “low” due to substantial heterogeneity. Survival rate was mainly rated “low” for most comparisons, attributable to both imprecision and heterogeneity. Most comparisons for CD3 + and CD4 + were rated “moderate,” although CCRT versus XAP + CCRT and CCRT versus YDZYR+CCRT were downgraded to “low” owing to imprecision and heterogeneity, respectively. All evidence for CD8 + and CD4+/CD8 + was rated “low,” reflecting imprecision or heterogeneity. Detailed CINeMA assessment results are provided in Supplementary material 9.

## 4 Discussion

This is the first NMA comparing the efficacy and safety of various CMIs combined with CCRT in the treatment of EC. A total of 54 eligible RCTs were included in the meta-analysis. Our results indicate that HQDT+CCRT is the most effective regimen for enhancing the clinical effectiveness rate; KLT + CCRT is most effective for improving performance status and the one-year survival rate; KA + CCRT demonstrates the greatest efficacy in increasing CD3 + levels; AD+CCRT is most effective in raising CD4 + and CD8 + levels; and FFKS+CCRT is the optimal regimen for enhancing the CD4+/CD8 + ratio. All treatment regimens showed favorable safety profiles, with no serious AEs reported. Regarding clinical effectiveness, APS exhibits a notable advantage. APS, the active component of Astragalus membranaceus, primarily exerts anticancer effects via immune activation, promotion of tumor cell apoptosis, and inhibition of lipid metabolism (100). APS can upregulate expression of TP73 and FBXW7 proteins, while downregulating Ki67 expression, thereby effectively inhibiting EC cell proliferation, with this inhibitory effect being dose-dependent (101). Additionally, APS promotes autophagy in EC109 cells by increasing Beclin1 and LC3 expression and decreasing the protein levels of P62 (102). Regulation of cytokine and chemokine expression is critical for alleviating the inflammatory state of tumors (103, 104). Sun et al. (105) demonstrated that a 7-day preoperative injection of APS (1 mg/kg, once daily) significantly reduced serum levels of IL-6, IL-12, and VEGF in EC patients, potentially mediated via the p-AKT signaling pathway. Chen et al. (106) further reported that APS significantly decreased PI3K and Akt expression in EC rats, with tumor inhibition rates of 45.59% (400 mg/kg), 32.35% (200 mg/kg), and 17.65% (100 mg/kg) under different dosing regimens. A randomized open-label clinical trial evaluating the combination of APS with CCRT for locally advanced EC is currently underway (107). Notably, although APS ranked highest for improving clinical effectiveness, only one study is available; thus, its ranking should be interpreted cautiously, and clinical use should consider multiple factors to maximize therapeutic benefit.

Approximately 60 to 80% of patients with EC experience malnutrition, weight loss, and cachexia, which significantly impair their quality of life and survival rates (108). KLT has demonstrated significant benefits in improving performance status and the one-year survival rate. Its main component, Coix seed oil, derived from *Coix lacryma-jobi* L (Poaceae), exhibits spleen-strengthening, dampness-resolving, and detoxifying effects, enhancing immune function and significantly improving patient quality of life (109, 110). Liu et al. (111) observed that oral administration of Coix seed oil (2.5 mL·kg^−1^·d^−1^) in cachectic mice markedly reduced weight loss, ameliorated muscle and fat atrophy, and did not affect food intake or tumor burden. Coix seed oil reduced muscle protein degradation and excessive lipolysis by lowering HSL phosphorylation in the AMPK signaling pathway and suppressing MuRF1 expression in the NF-κB pathway. These findings suggest potential long-term benefits in improving quality of life, warranting further investigation. In triple-negative breast cancer models, KLT effectively blocked cell cycle progression at the G2/M phase by downregulating CDK1, CDK2, and CHEK1, inhibiting CDC25A, CDC25B, MELK, and AURKA activity, suppressing mitosis, and inducing apoptosis (112). In terms of adjuvant therapy, KLT increases cancer cell sensitivity to chemotherapeutics via JAK2/STAT3 and NF-κB pathway modulation, downregulating MDR1, MRP1, and PVT1, while mitigating chemotherapy-related adverse effects. It has been widely applied in liver, gastric, NSCLC, and colorectal cancers (113–116).

KA shows significant efficacy in enhancing CD3 + T lymphocyte levels. Composed of extracts from Ginseng Radix et Rhizoma, Sophorae Flavescentis Radix, and Astragali Radix, it contains 11 alkaloids, 8 astragalosides, and 28 ginsenosides (117). Pharmacological studies have shown that Astragalus enhances immune function, mitigates myocardial ischemia–reperfusion injury, and possesses multiple pharmacological actions, including anti-inflammatory, antioxidant, and anti-tumor effects (118, 119). Ginsenosides from Ginseng modulate T lymphocyte subsets, improving cellular immunity and conferring anti-fatigue, anti-aging, and neuroprotective effects (120–122). Sophora has demonstrated excellent antiviral activity and liver-protective effects (123). The synergistic effects of KA combined with radiotherapy or chemotherapy for EC have been validated clinically, with underlying mechanisms under investigation (124–127). Li et al. (128) used network pharmacology to identify 87 active ingredients, 172 potential therapeutic targets for EC, and the major implicated PI3K/AKT pathway in KA. Cell experiments further confirmed that the primary components, Astragaloside IV and Ginsenoside Rk3, demonstrate anti-EC effects through the suppression of the PI3K-AKT signaling pathway (129, 130). Pharmacokinetic studies revealed that the terminal elimination half-life (t_1/2_) of Oxymatrine, the index component of KA, in rat plasma was 2.73 ± 1.16 h, with a cumulative maximum concentration (C_max_) of 422.70 ± 55.50 nmol·L^−1^, total plasma clearance (CL_tot_) of 111.34 ± 18.49 mL·h^−1^·kg^−1^, area under the concentration-time curve (AUC_0-t_) of 502.71 ± 93.02 nmol·L^−1^·h^−1^, and steady-state volume of distribution (V_ss_) of 220.11 ± 53.82 mL·kg^−1^. Additionally, studies have shown that KA exhibits weak inhibition of major drug-metabolizing enzymes, CYP and UGT isoenzymes, and is unlikely to cause significant drug–drug interactions (DDIs), which enhances its clinical safety and convenience (131).

AD demonstrates significant efficacy in enhancing CD4 + and CD8 + T cell counts in EC patients. Primarily composed of ginseng, eleutherococcus, astragalus, and cantharidin, AD exerts dual effects of tonifying qi and augmenting vital energy while simultaneously expelling pathogenic factors and detoxifying, particularly suitable for EC patients with qi-deficiency and toxin-stasis patterns presenting with fatigue, dysphagia, and dark purple tongue (132). As a classical TCM formulation, AD exhibits notable anticancer activity *in vitro* and *in vivo* against gastrointestinal tumors. Lu et al. (133) reported that AD targets BIRC5 and FEN1, genes closely linked to immune modulation, producing substantial anticancer effects in HCC patients via the combined action of cantharidin, formononetin, and isofraxidin. Furthermore, AD regulates the Th1/Th2 immune balance in advanced colorectal cancer sufferers, increasing serum levels of prealbumin, IgA, and IgG, thereby effectively improving the patient’s immune status (134, 135). The meta-analysis by Huang et al. (28) proved that AD in combination with radiochemotherapy significantly improves objective response rate and functional status, and reduces bone marrow suppression (BMS), chemotherapy-induced nausea and vomiting (CINV), and radiation esophagitis (RE) in patients with unresectable EC.

Interestingly, reductions in CD8 + T cell counts following AD treatment were associated with improved prognosis, which may reflect its immune-regulatory effects. Prolonged antitumor immune responses can drive CD8 + T cells toward functional exhaustion, impairing cytotoxic efficacy, potentially influenced by tumor microenvironment (TME) alterations, immunosuppressive mechanisms, and tumor immune evasion (136, 137). Post-treatment reduction of dysfunctional CD8 + T cells may facilitate the activation of other functionally competent immune cells, thereby improving overall immune status. Shi et al. (138) demonstrated that AD suppresses EC cell invasiveness and migration by inhibiting EMT signaling and VEGF expression. Notably, modulating EMT signaling may impact cancer-associated fibroblasts (CAFs) in the TME, improve T cell function, and enhance immune surveillance (139). The underlying mechanisms may involve inhibition of CAF activation, reduction of TGF-*β* secretion, and decreased aggregation of regulatory T cells (Tregs) and myeloid-derived suppressor cells (MDSCs), thereby diminishing recruitment and infiltration of immunosuppressive cells, and creating a more favorable TME for CD8 + T cells (140, 141). Additionally, modulation of the EMT signaling pathway may alter intercellular communication and cytokine networks in the TME, promoting anti-tumor immune responses and enhancing the anti-tumor activity of T cells (140). Anti-PD-L1 therapy, which enhances T cell antitumor function by alleviating PD-1/PD-L1-mediated suppression, acts via a similar mechanism, highlighting that functional restoration of CD8 + T cells may be more critical than mere increases in cell number (142, 143). Additional quality research is required to validate these results.

FFKS demonstrates significant benefits in improving the CD4+/CD8 + ratio. FFKS comprises *Sophora flavescens* Aiton (Fabaceae) and Smilax glabra Roxb (Smilacaceae) (144). Research indicates that these two herbal components exhibit a notable synergistic effect in their anticancer properties (145). The primary component, *Sophora flavescens* Aiton, acts on various stages of the cell cycle, effectively inducing apoptosis and inhibiting tumor cells in the G0, G1, S, G2, and M phases, while further blocking cancer cell growth by suppressing energy metabolism and DNA repair pathways (146–148). Its primary alkaloid, matrine, modulates dendritic cell (DC) maturation by reducing ROS, activating ERK1/2 signaling, and inhibiting NF-κB, thereby regulating CD4 + and CD8 + T cell proliferation, increasing Treg proportions, and significantly affecting the CD4+/CD8 + ratio (149). Although Smilax glabra Roxb contains relatively fewer chemical components, its combination with *Sophora flavescens* Aiton not only enhances the cytotoxic effects against cancer cells but also strengthens the body’s immune response to tumors by upregulating Interleukin-1β expression (145). Zhu et al. (150) reported that nude mice inoculated with EC9706 cells and administered 200 μL/d of FFKS intraperitoneally for 4 weeks exhibited reduced PCNA and Bcl-2 expression and a tumor inhibition rate of 49%, likely mediated by caspase-3 activation and Fas upregulation. Moreover, Zhou et al. (151), through WGCNA analysis combined with network pharmacology methods, identified ErbB2, CCND1, and IGF1R as potential targets of FFKS for EC therapy. Pharmacokinetic studies in rats demonstrated the t_1/2_ of FFKS of 1.449 ± 0.496 h, C_max_ of 2.032 ± 7.151 μg/mL, AUC_0-t_ of 7,397 ± 2,082 ng·mL^−1^·h^−1^, volume of distribution during elimination (V_z_) of 1.171 ± 0.422 L·kg^−1^, and terminal clearance (CL_z_) of 0.579 ± 0.179 L·h^−1^·kg^−1^. These results suggest that FFKS exhibits favorable pharmacokinetic properties in nude mice (152).

### 4.1 Limitations

Firstly, there existed geographical limitations. Although an extensive search was performed across eight databases, the included RCTs primarily involved Chinese populations. Consequently, the generalizability of our findings to other regions or populations remains uncertain. Clinical applications should carefully consider population characteristics and regional variations in medical practice. Secondly, the number of studies for certain CMIs was limited, particularly HQDT, SQFZ, SF, and XAP, for which only one RCT was available, reducing the reliability of these results. Further pharmacological studies and high-quality RCTs are therefore required to substantiate these findings. Thirdly, long-term data were lacking. This study mainly focused on short-term outcomes, including survival rates and quality of life, without fully addressing the long-term prognosis of patients with EC. Future research should place greater emphasis on long-term efficacy and clinically meaningful outcomes. Fourthly, limitations in study design were evident. Some RCTs lacked effective blinding, potentially introducing bias. Additionally, subgroup analyses were not feasible due to insufficient data, further affecting the robustness of the results. It should be noted that, although our study suggests potential efficacy advantages of specific CMIs combined with CCRT, CINeMA assessment indicated that the quality of evidence for all outcomes was only low to moderate. Therefore, current findings are insufficient to form strong clinical recommendations, and practical application should integrate individual patient characteristics. Further verification through rigorously designed, multicenter RCTs is needed.

## 5 Conclusion

Our Bayesian NMA demonstrated that CMIs combined with CCRT significantly improve the health status of patients with EC, reduce toxic side effects, and enhance quality of life. Among the CMIs, HQDT, KLT, KA, AD, and FFKS, when combined with CCRT, showed potential as preferred treatment options for EC. Notably, although HQDT ranked highest in clinical effectiveness, this finding is based on a single RCT and requires further validation. Given that CINeMA rated the overall evidence quality as low to moderate, future high-quality, large-scale, double-blind RCTs are needed to confirm these conclusions.

## Data Availability

The original contributions presented in the study are included in the article/Supplementary material, further inquiries can be directed to the corresponding author/s.
