# Supplementary material for: Efficacy and safety of Chinese medicine injection combined with concurrent chemoradiotherapy in the treatment of esophageal cancer: a Bayesian network meta-analysis
Source: Front Med (Lausanne). 2025 Oct 14;12:1643598. doi: 10.3389/fmed.2025.1643598 (PMC12558960; doi:10.3389/fmed.2025.1643598)
Supplement: Supplementary file 5 [file Data_Sheet_5.docx]

**Global Consistency Analysis**

| Outcomes | Clinical effectiveness rate | Performance status | Survival rate | CD3+ | CD4+ | CD8+ | CD4+/CD8+ |
| --- | --- | --- | --- | --- | --- | --- | --- |
| Consistency model DIC | 158.67159 | 33.70064 | 36.44581 | 27.83613 | 47.75793 | 42.91057 | 23.88400 |
| Inconsistency model DIC | 158.62521 | 33.53004 | 36.64095 | 27.81263 | 47.79110 | 42.92776 | 23.87312 |
