# Supplementary material for: Efficacy and safety of Chinese medicine injection combined with concurrent chemoradiotherapy in the treatment of esophageal cancer: a Bayesian network meta-analysis
Source: Front Med (Lausanne). 2025 Oct 14;12:1643598. doi: 10.3389/fmed.2025.1643598 (PMC12558960; doi:10.3389/fmed.2025.1643598)

**Convergence Diagnosis of Outcomes**

Fig 1. Convergence Diagnosis for clinical effectiveness rate


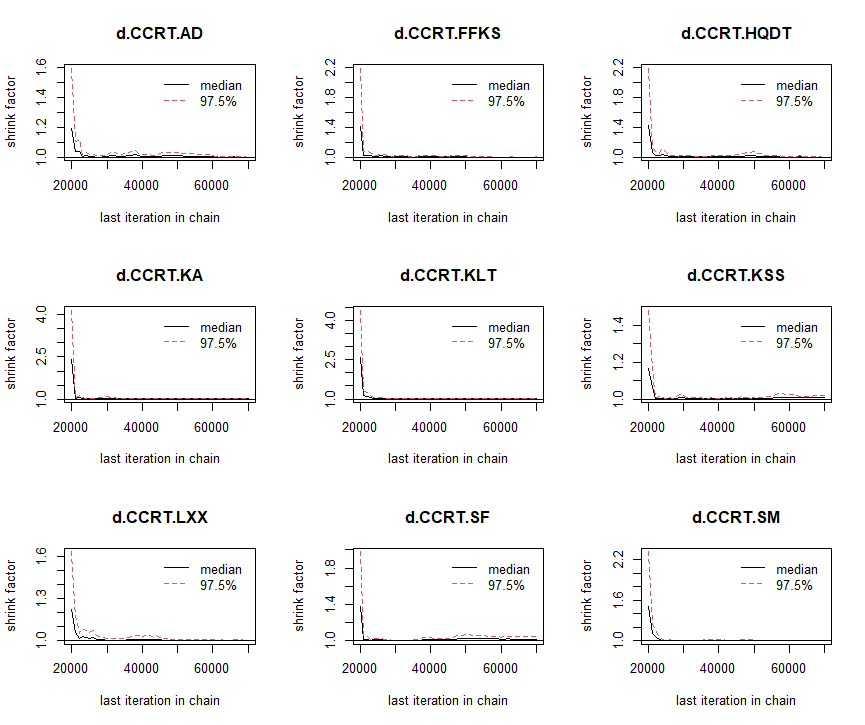


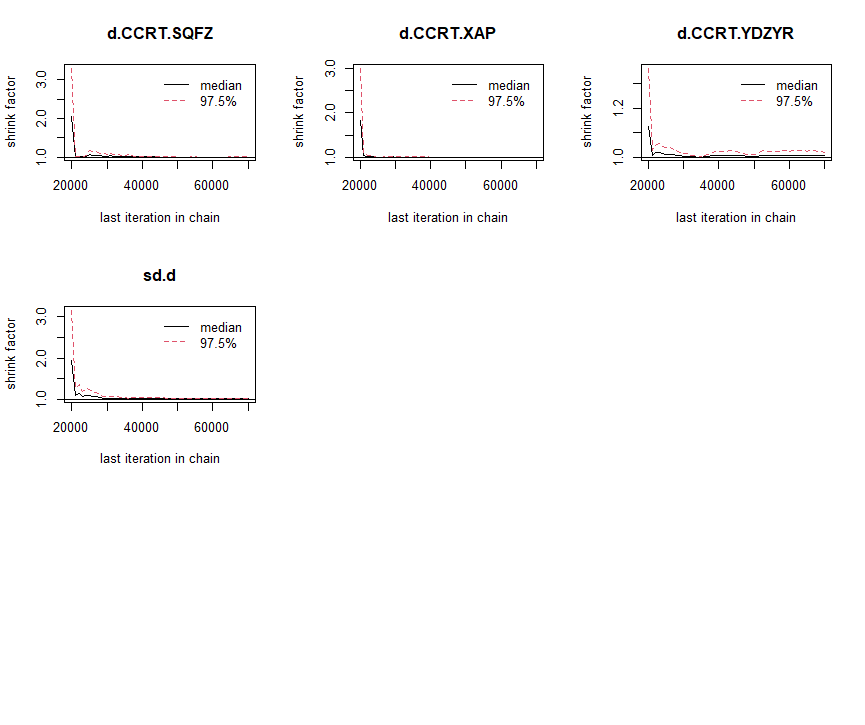


Fig2. Convergence Diagnosis for performance status


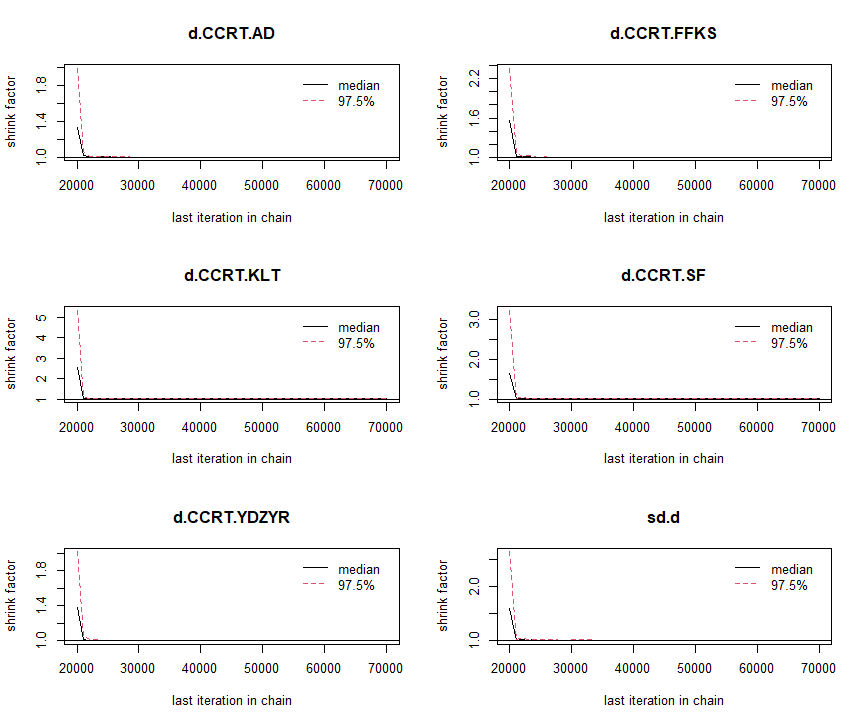


Fig3. Convergence Diagnosis for survival rate


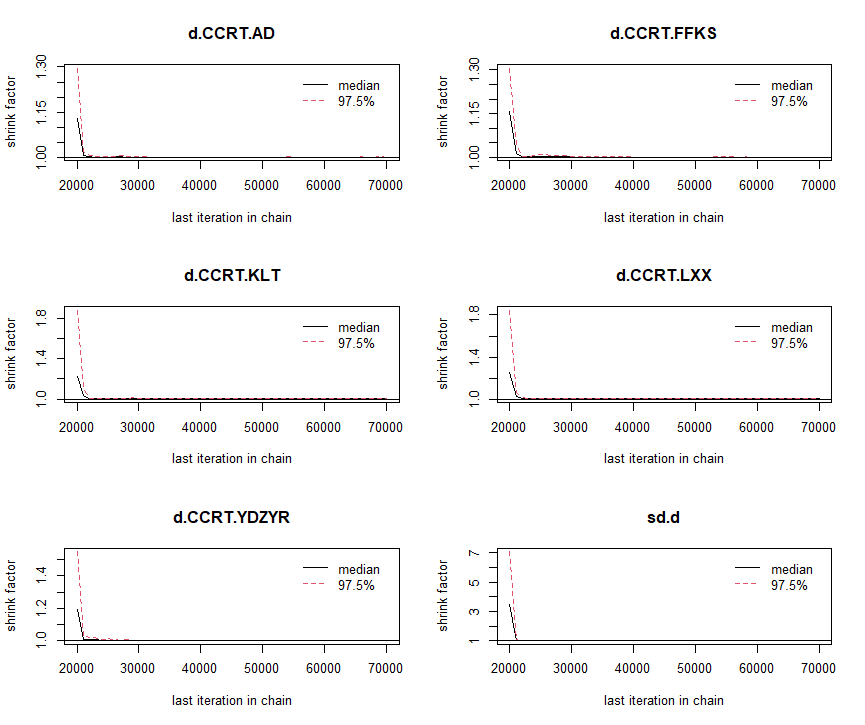


Fig4. Convergence Diagnosis for CD3+


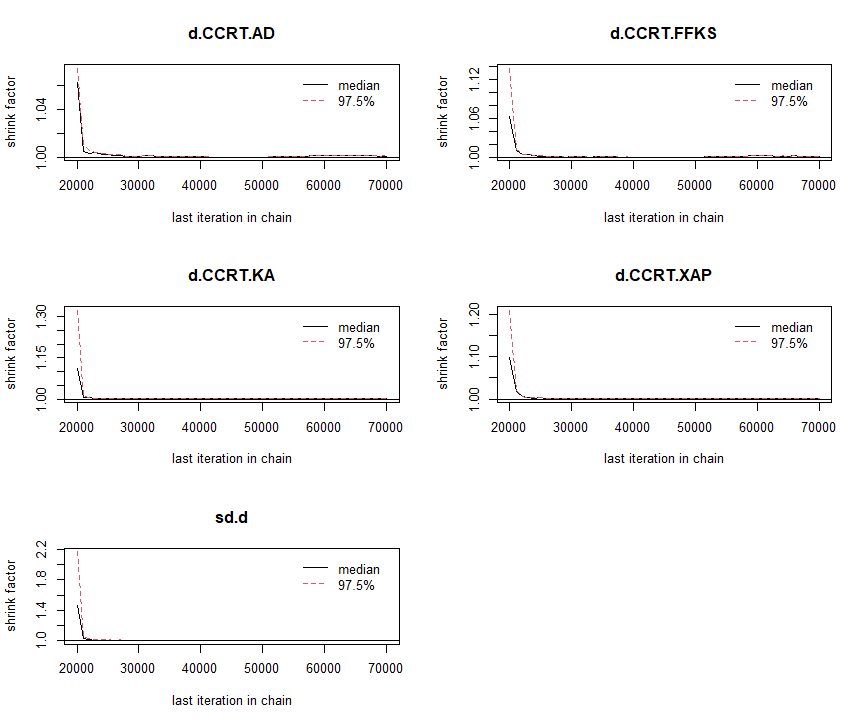


Fig5. Convergence Diagnosis for CD4+


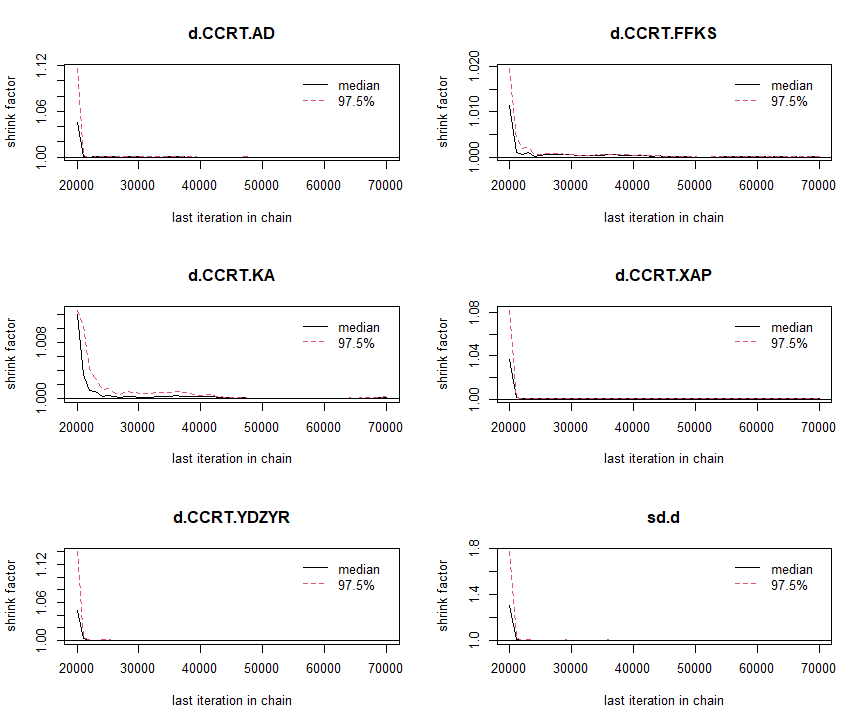


Fig6. Convergence Diagnosis for CD8+


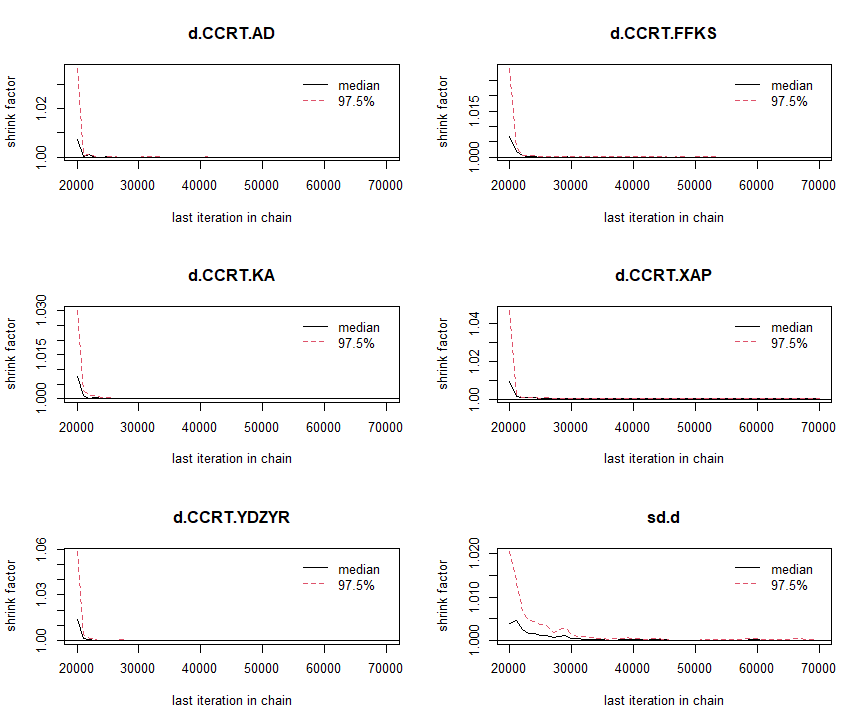


Fig7. Convergence Diagnosis for CD4+/CD8+


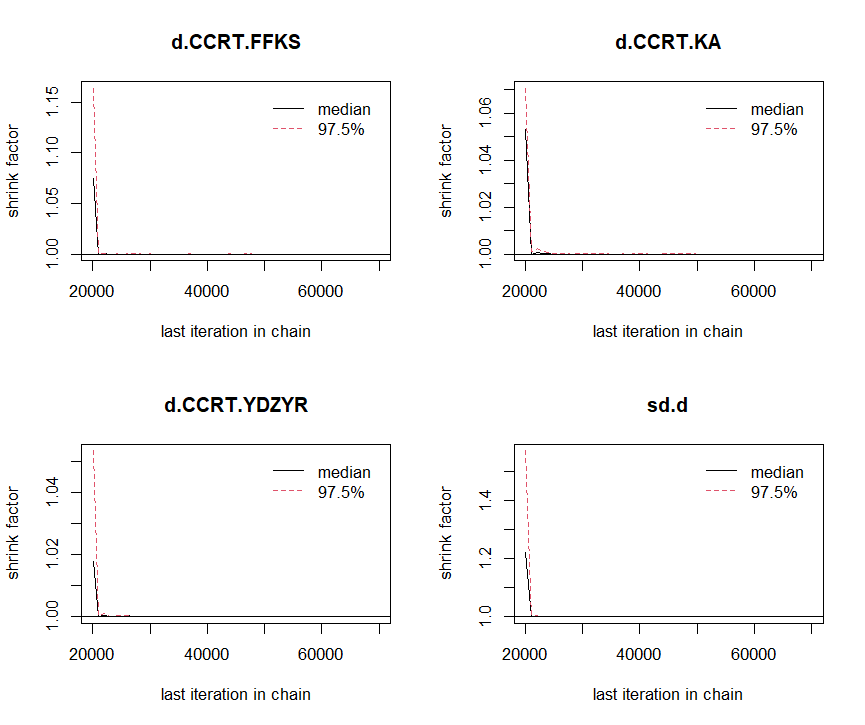

Supplement: Supplementary file 6 [file Data_Sheet_6.docx]
