# Supplementary material for: Efficacy and safety of Chinese medicine injection combined with concurrent chemoradiotherapy in the treatment of esophageal cancer: a Bayesian network meta-analysis
Source: Front Med (Lausanne). 2025 Oct 14;12:1643598. doi: 10.3389/fmed.2025.1643598 (PMC12558960; doi:10.3389/fmed.2025.1643598)

**Heterogeneity analysis of Outcomes**

Fig 1. Heterogeneity analysis of clinical effectiveness rate


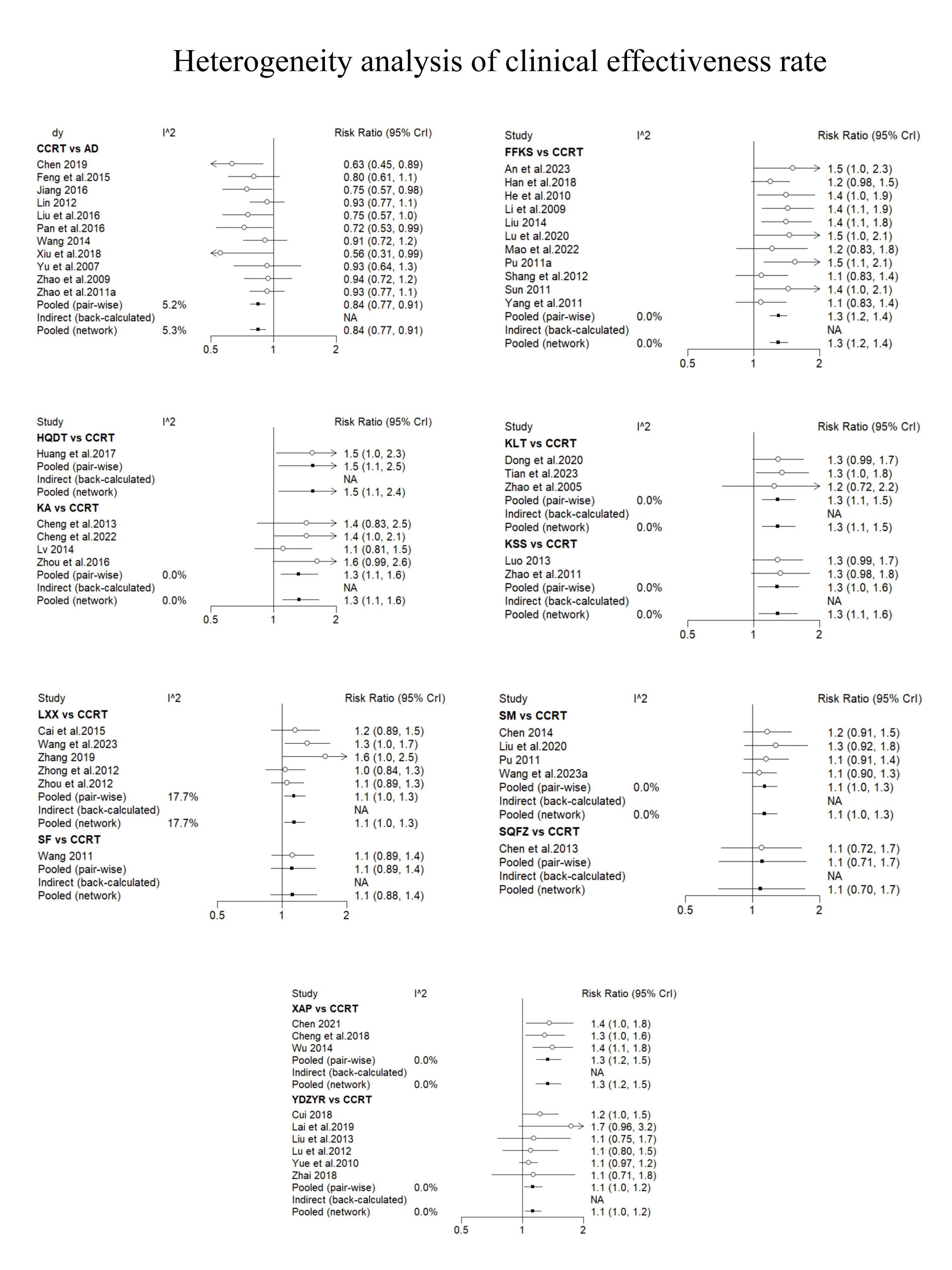


Fig 2. Heterogeneity analysis of performance status


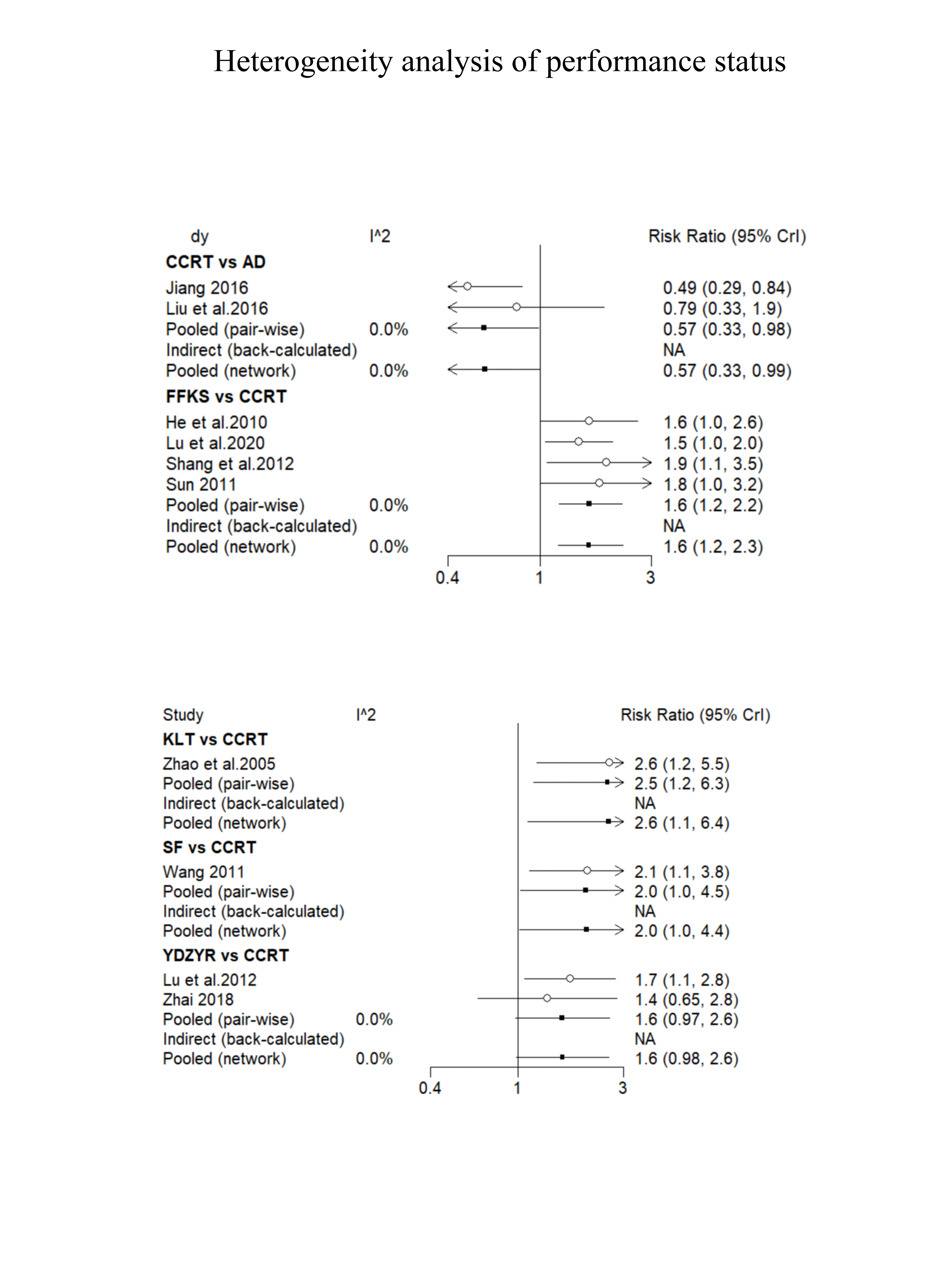


Fig 3. Heterogeneity analysis of 1 year survival rate


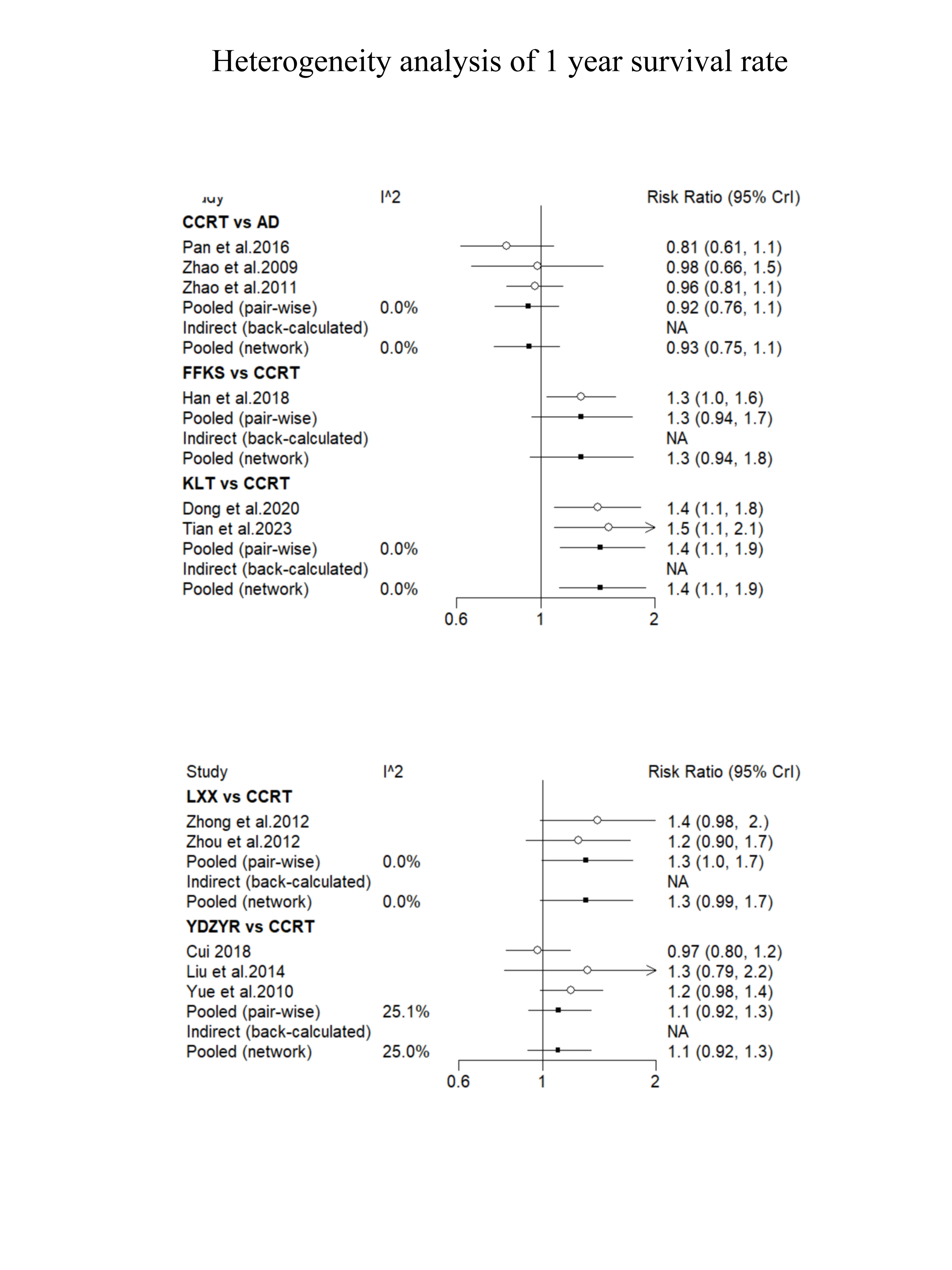


Fig 4. Heterogeneity analysis of CD3+


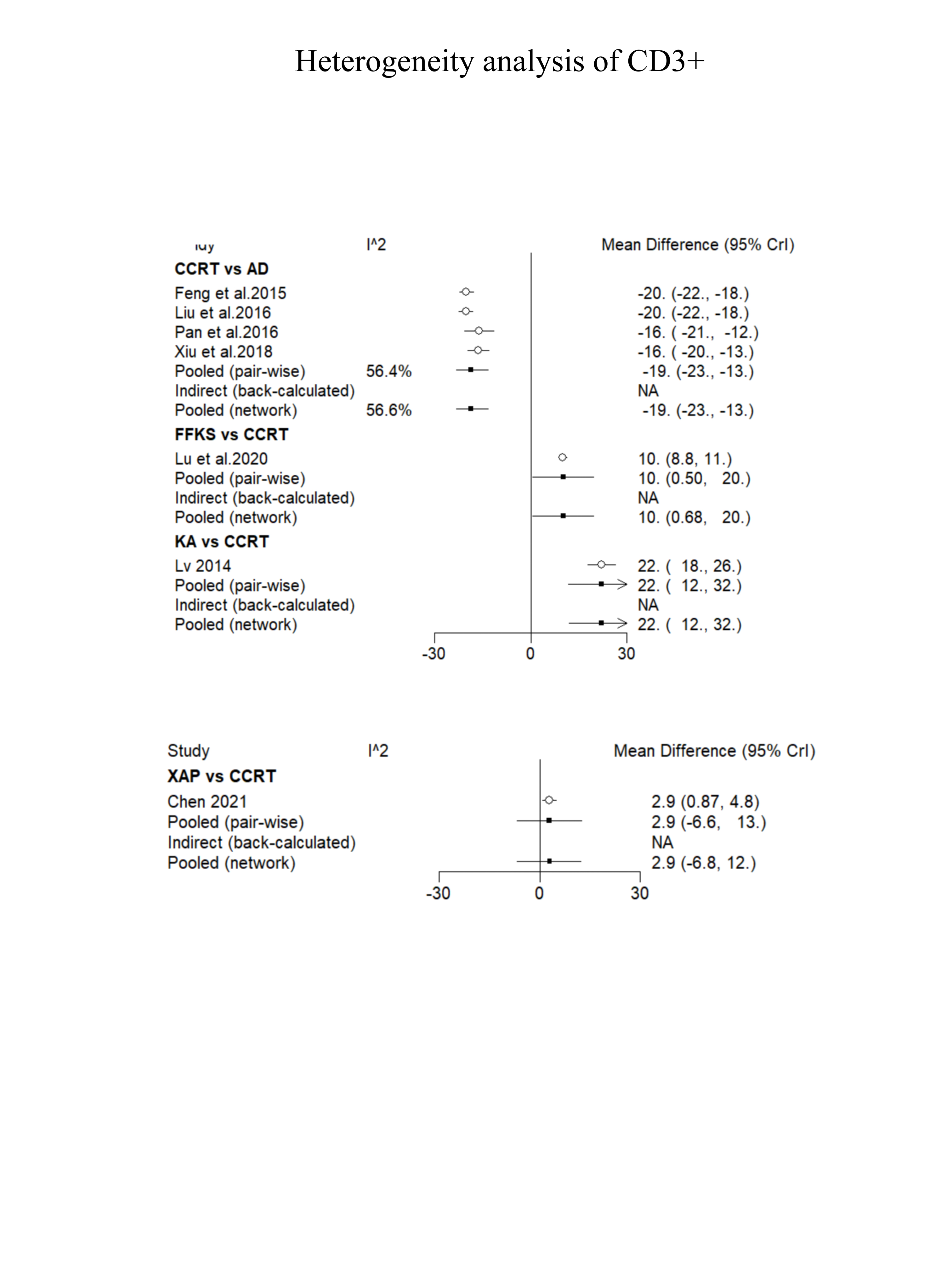


Fig 5. Heterogeneity analysis of CD4+


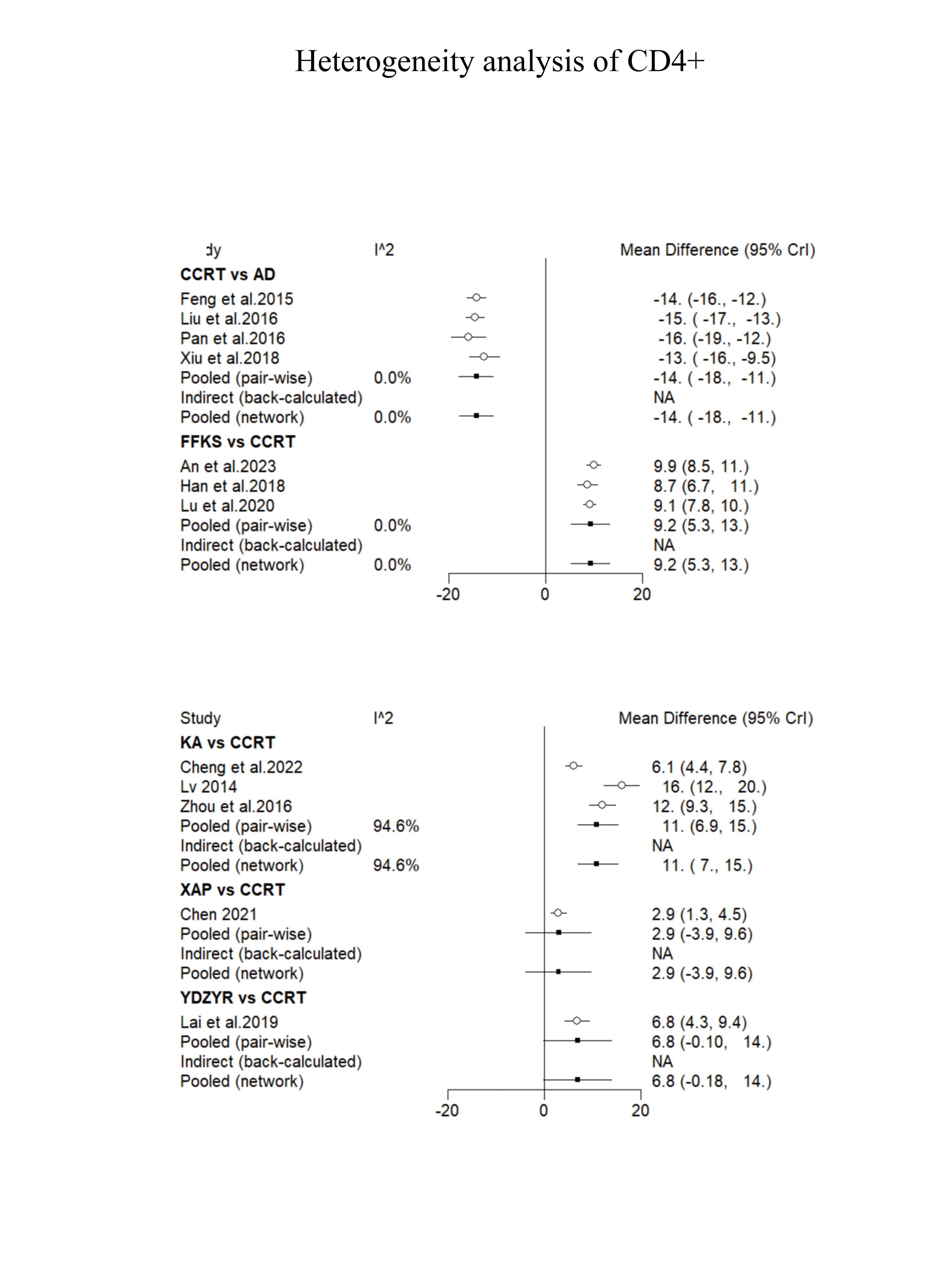


Fig 6. Heterogeneity analysis of CD8+


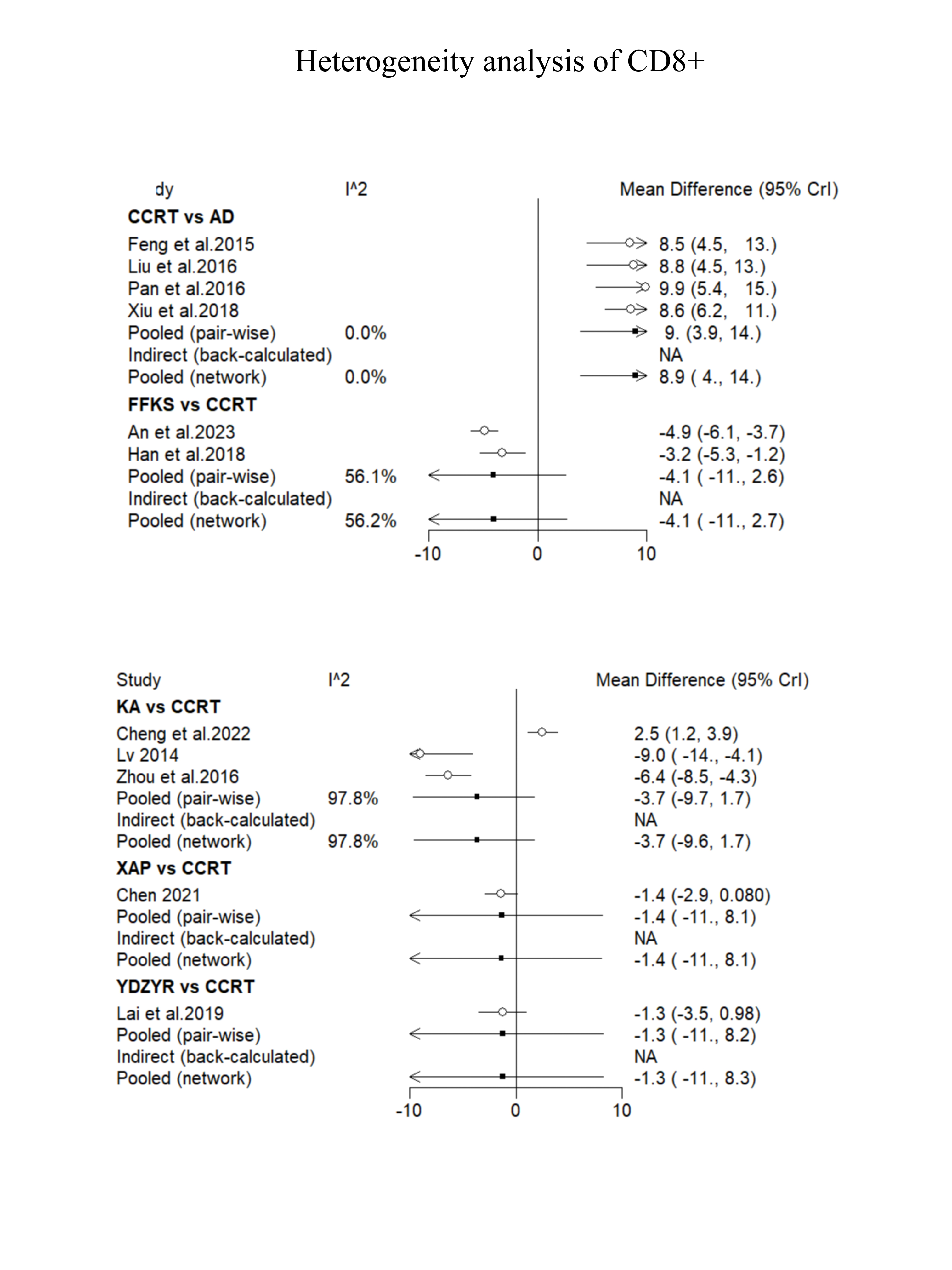


Fig 7. Heterogeneity analysis of CD4+/CD8+


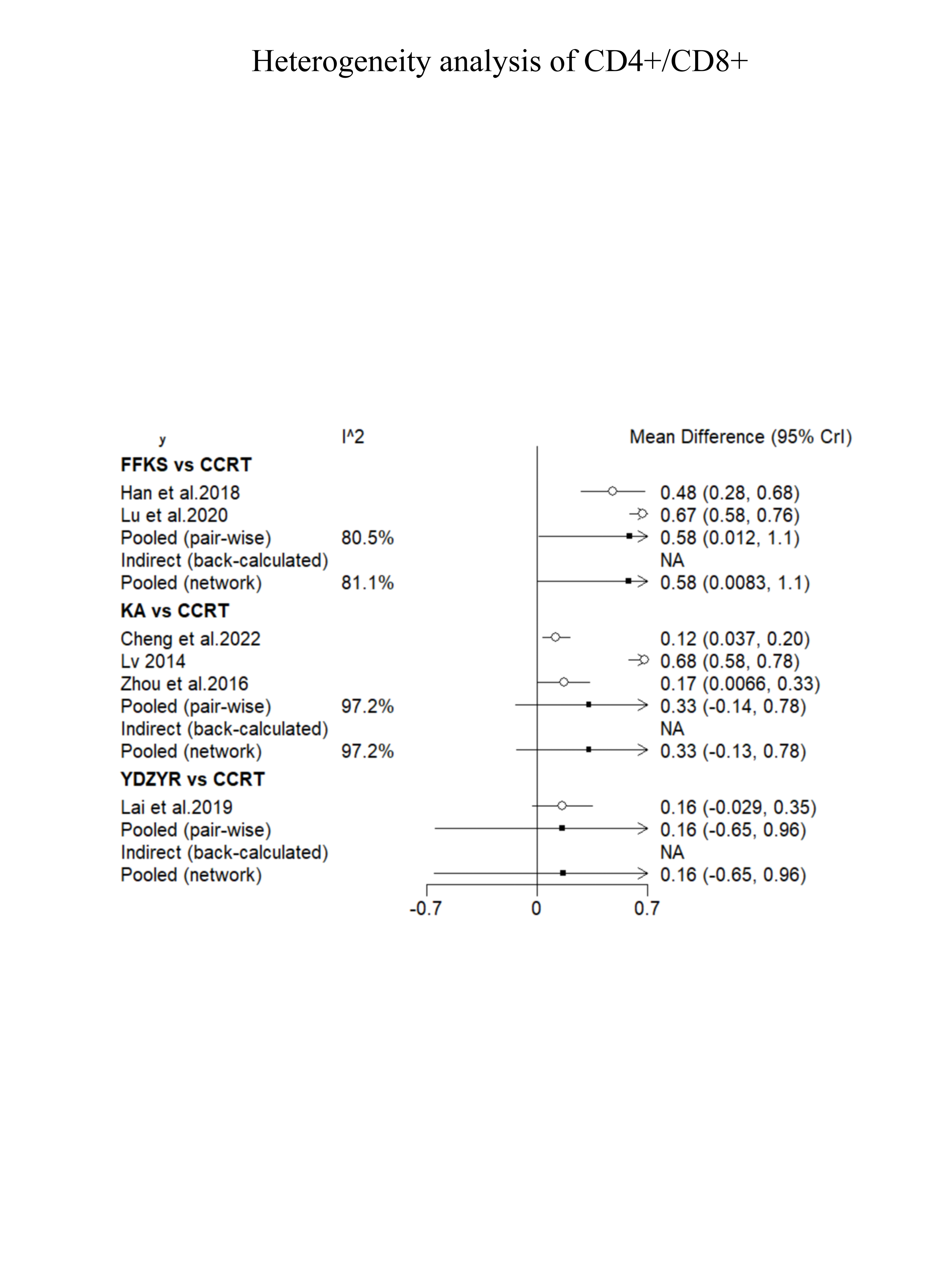

Supplement: Supplementary file 7 [file Data_Sheet_7.docx]
