# Supplementary material for: Efficacy and safety of Chinese medicine injection combined with concurrent chemoradiotherapy in the treatment of esophageal cancer: a Bayesian network meta-analysis
Source: Front Med (Lausanne). 2025 Oct 14;12:1643598. doi: 10.3389/fmed.2025.1643598 (PMC12558960; doi:10.3389/fmed.2025.1643598)

**Egger’s test**

| Outcome | Number of studies | Egger’s test *p* value |
| --- | --- | --- |
|  |  |  |
| 3.4.2 Clinical effectiveness rate | 52 | *P* = 0.082 |
| 3.4.3 Performance status | 10 | *P* = 0.942 |
| 3.4.4 Survival rate | 11 | *P* = 0.209 |
| 3.4.5 CD3+ | 7 | *P* = 0.409 |
| 3.4.6 CD4+ | 12 | *P* = 0.340 |
| 3.4.7 CD8+ | 11 | *P* = 0.237 |
| 3.4.8 CD4+/CD8+ | 6 | *P* = 0.731 |

Egger’s plot

3.4.2 Clinical effectiveness rate


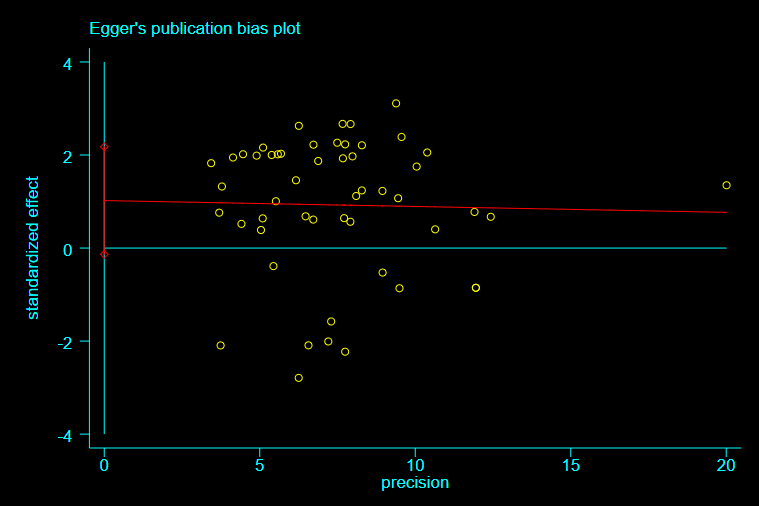


3.4.3 Performance status


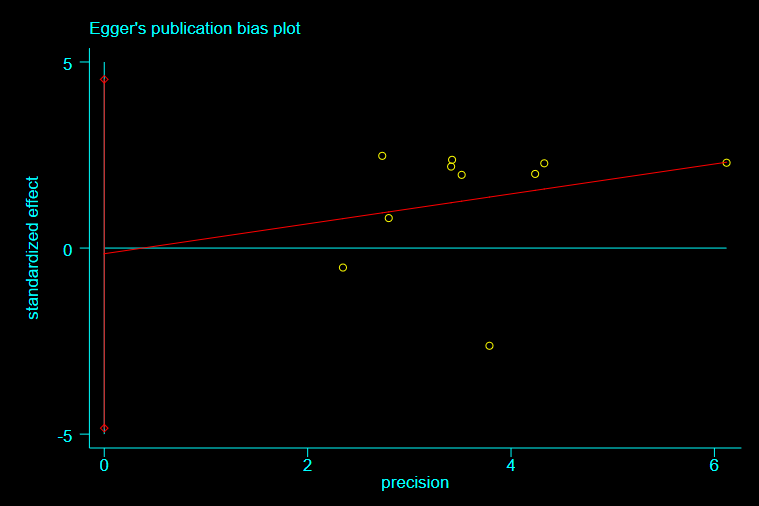


3.4.4 Survival rate


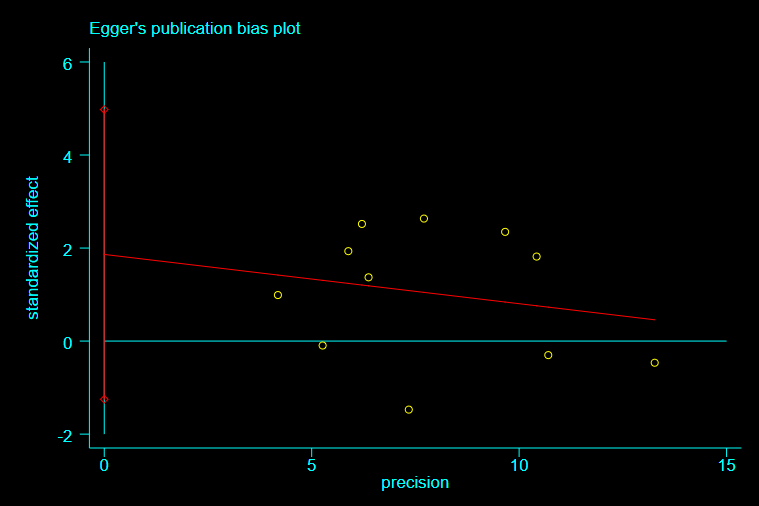


3.4.5 CD3+


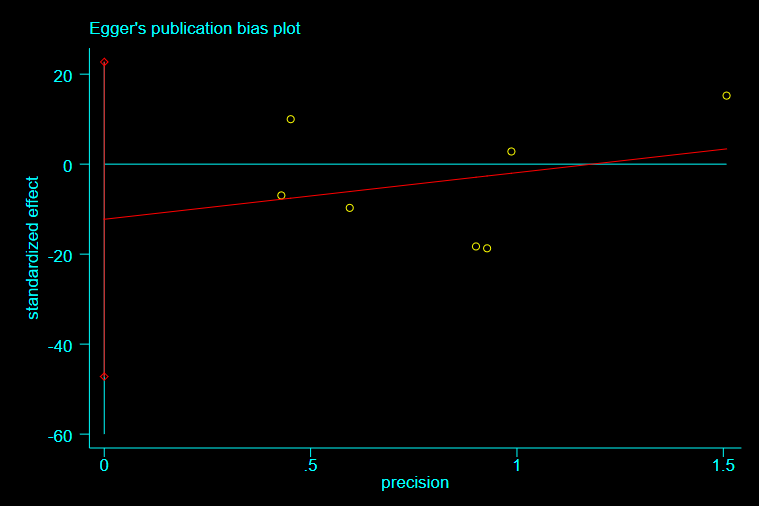


3.4.6 CD4+


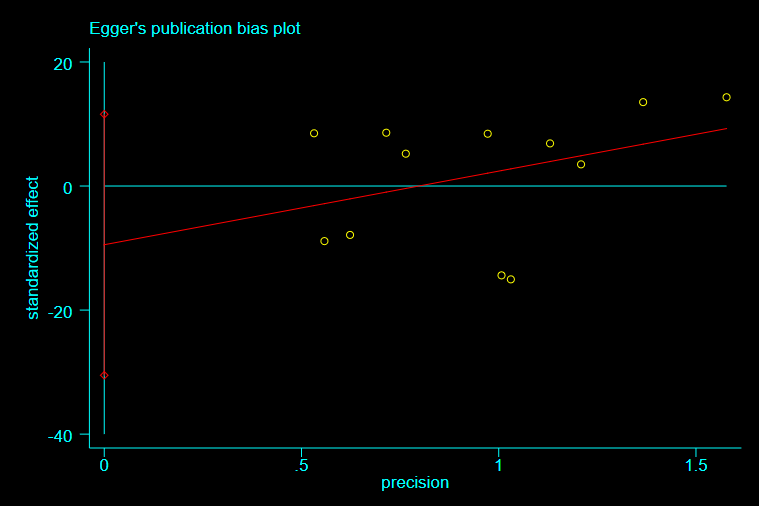


3.4.7 CD8+


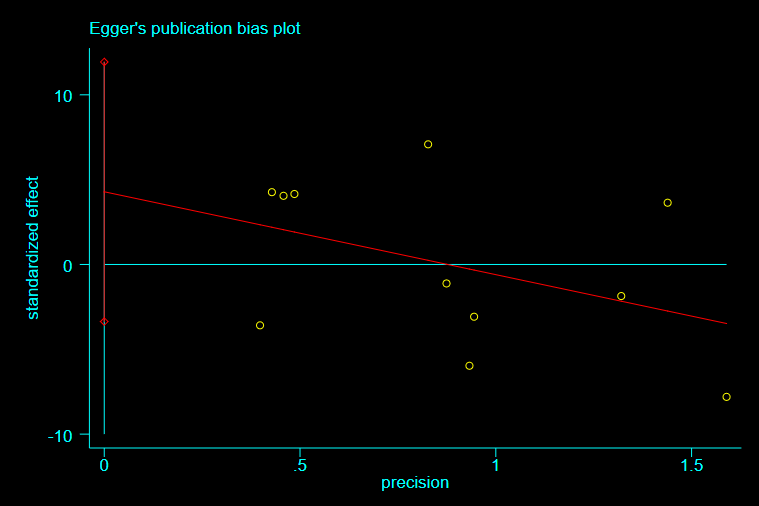


3.4.8 CD4+/CD8+


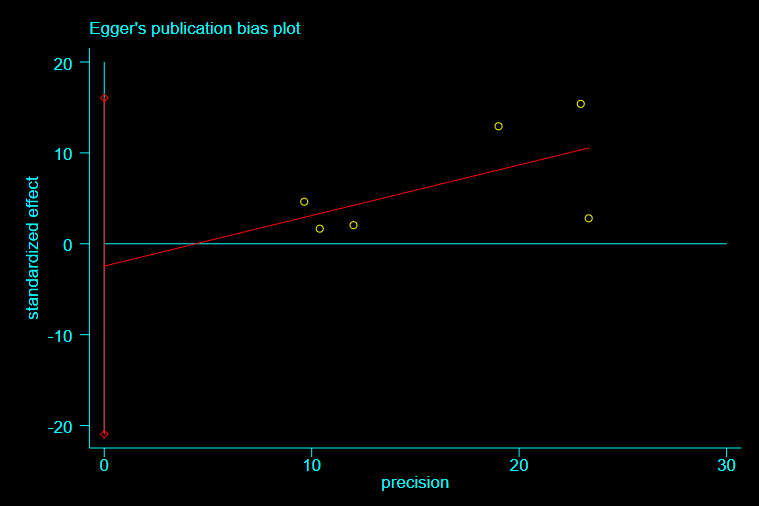

Supplement: Supplementary file 8 [file Data_Sheet_8.docx]
