# Supplementary material for: Efficacy and safety of Chinese medicine injection combined with concurrent chemoradiotherapy in the treatment of esophageal cancer: a Bayesian network meta-analysis
Source: Front Med (Lausanne). 2025 Oct 14;12:1643598. doi: 10.3389/fmed.2025.1643598 (PMC12558960; doi:10.3389/fmed.2025.1643598)
Supplement: Supplementary file 9 [file Data_Sheet_9.docx]

**Table1: Confidence rating table for clinical effectiveness rate**

| Comparison | Number of studies | Within-study bias | Reporting bias | Indirectness | Imprecision | Heterogeneity | Incoherence | Confidence rating |
| --- | --- | --- | --- | --- | --- | --- | --- | --- |
| CCRT:AD+CCRT | 11 | Some concerns | Low risk | No concerns | No concerns | No concerns | Some concerns | Moderate |
| CCRT:FFKS+CCRT | 11 | Some concerns | Low risk | No concerns | No concerns | No concerns | Some concerns | Moderate |
| CCRT:HQDT+CCRT | 1 | Some concerns | Low risk | No concerns | No concerns | No concerns | Some concerns | Moderate |
| CCRT:KA+CCRT | 4 | Some concerns | Low risk | No concerns | No concerns | No concerns | Some concerns | Moderate |
| CCRT:KLT+CCRT | 3 | Some concerns | Low risk | No concerns | No concerns | No concerns | Some concerns | Moderate |
| CCRT:KSS+CCRT | 2 | Some concerns | Low risk | No concerns | No concerns | No concerns | Some concerns | Moderate |
| CCRT:LXX+CCRT | 5 | Some concerns | Low risk | No concerns | No concerns | No concerns | Some concerns | Moderate |
| CCRT:SF+CCRT | 1 | Some concerns | Low risk | No concerns | Major concerns | No concerns | Some concerns | Low |
| CCRT:SM+CCRT | 4 | Some concerns | Low risk | No concerns | No concerns | No concerns | Some concerns | Moderate |
| CCRT:SQFZ+CCRT | 1 | Some concerns | Low risk | No concerns | Major concerns | No concerns | Some concerns | Low |
| CCRT:XAP+CCRT | 3 | Some concerns | Low risk | No concerns | No concerns | No concerns | Some concerns | Moderate |
| CCRT:YDZYR+CCRT | 6 | Some concerns | Low risk | No concerns | No concerns | No concerns | Some concerns | Moderate |

**Table2: Confidence rating table for performance status**

| Comparison | Number of studies | Within-study bias | Reporting bias | Indirectness | Imprecision | Heterogeneity | Incoherence | Confidence rating |
| --- | --- | --- | --- | --- | --- | --- | --- | --- |
| CCRT:AD+CCRT | 2 | Some concerns | Low risk | No concerns | No concerns | Major concerns | Some concerns | Low |
| CCRT:FFKS+CCRT | 4 | No concerns | Low risk | No concerns | No concerns | No concerns | Some concerns | Moderate |
| CCRT:KLT+CCRT | 1 | Some concerns | Low risk | No concerns | No concerns | Major concerns | Some concerns | Low |
| CCRT:SF+CCRT | 1 | Some concerns | Low risk | No concerns | No concerns | Major concerns | Some concerns | Low |
| CCRT:YDZYR+CCRT | 2 | Some concerns | Low risk | No concerns | No concerns | Major concerns | Some concerns | Low |

**Table3: Confidence rating table for survival rate**

| Comparison | Number of studies | Within-study bias | Reporting bias | Indirectness | Imprecision | Heterogeneity | Incoherence | Confidence rating |
| --- | --- | --- | --- | --- | --- | --- | --- | --- |
| CCRT:AD+CCRT | 3 | Some concerns | Low risk | No concerns | Major concerns | No concerns | Some concerns | Low |
| CCRT:FFKS+CCRT | 1 | Some concerns | Low risk | No concerns | No concerns | Major concerns | Some concerns | Low |
| CCRT:KLT+CCRT | 2 | Some concerns | Low risk | No concerns | No concerns | No concerns | Some concerns | Moderate |
| CCRT:LXX+CCRT | 2 | Some concerns | Low risk | No concerns | No concerns | Major concerns | Some concerns | Low |
| CCRT:YDZYR+CCRT | 3 | Some concerns | Low risk | No concerns | Major concerns | No concerns | Some concerns | Low |

**Table4: Confidence rating table for CD3+**

| Comparison | Number of studies | Within-study bias | Reporting bias | Indirectness | Imprecision | Heterogeneity | Incoherence | Confidence rating |
| --- | --- | --- | --- | --- | --- | --- | --- | --- |
| CCRT:AD+CCRT | 4 | Some concerns | Low risk | No concerns | No concerns | No concerns | Some concerns | Moderate |
| CCRT:FFKS+CCRT | 1 | Some concerns | Low risk | No concerns | No concerns | No concerns | Some concerns | Moderate |
| CCRT:KA+CCRT | 1 | Some concerns | Low risk | No concerns | No concerns | No concerns | Some concerns | Moderate |
| CCRT:XAP+CCRT | 1 | Some concerns | Low risk | No concerns | Major concerns | No concerns | Some concerns | Low |

**Table5: Confidence rating table for CD4+**

| Comparison | Number of studies | Within-study bias | Reporting bias | Indirectness | Imprecision | Heterogeneity | Incoherence | Confidence rating |
| --- | --- | --- | --- | --- | --- | --- | --- | --- |
| CCRT:AD+CCRT | 4 | Some concerns | Low risk | No concerns | No concerns | No concerns | Some concerns | Moderate |
| CCRT:FFKS+CCRT | 3 | Some concerns | Low risk | No concerns | No concerns | No concerns | Some concerns | Moderate |
| CCRT:KA+CCRT | 3 | Some concerns | Low risk | No concerns | No concerns | No concerns | Some concerns | Moderate |
| CCRT:XAP+CCRT | 1 | Some concerns | Low risk | No concerns | Major concerns | No concerns | Some concerns | Low |
| CCRT:YDZYR+CCRT | 1 | Some concerns | Low risk | No concerns | No concerns | Major concerns | Some concerns | Low |

**Table6: Confidence rating table for CD8+**

| Comparison | Number of studies | Within-study bias | Reporting bias | Indirectness | Imprecision | Heterogeneity | Incoherence | Confidence rating |
| --- | --- | --- | --- | --- | --- | --- | --- | --- |
| CCRT:AD+CCRT | 4 | Some concerns | Low risk | No concerns | No concerns | Major concerns | Some concerns | Low |
| CCRT:FFKS+CCRT | 2 | Some concerns | Low risk | No concerns | Major concerns | No concerns | Some concerns | Low |
| CCRT:KA+CCRT | 3 | Some concerns | Low risk | No concerns | Major concerns | No concerns | Some concerns | Low |
| CCRT:XAP+CCRT | 1 | Some concerns | Low risk | No concerns | Major concerns | No concerns | Some concerns | Low |
| CCRT:YDZYR+CCRT | 1 | Some concerns | Low risk | No concerns | Major concerns | No concerns | Some concerns | Low |

**Table7: Confidence rating table for CD4+/CD8+**

| Comparison | Number of studies | Within-study bias | Reporting bias | Indirectness | Imprecision | Heterogeneity | Incoherence | Confidence rating |
| --- | --- | --- | --- | --- | --- | --- | --- | --- |
| CCRT:FFKS+CCRT | 2 | Some concerns | Low risk | No concerns | No concerns | Major concerns | Some concerns | Low |
| CCRT:KA+CCRT | 3 | Some concerns | Low risk | No concerns | Major concerns | No concerns | Some concerns | Low |
| CCRT:YDZYR+CCRT | 1 | Some concerns | Low risk | No concerns | Major concerns | No concerns | Some concerns | Low |
